# Transcriptional and Non-Transcriptional Activation, Posttranslational Modifications, and Antiviral Functions of Interferon Regulatory Factor 3 and Viral Antagonism by the SARS-Coronavirus

**DOI:** 10.3390/v13040575

**Published:** 2021-03-29

**Authors:** Anna Glanz, Sukanya Chakravarty, Merina Varghese, Anita Kottapalli, Shumin Fan, Ritu Chakravarti, Saurabh Chattopadhyay

**Affiliations:** 1 Department of Medical Microbiology and Immunology, University of Toledo College of Medicine and Life Sciences, Toledo, OH 43614, USA; Anna.Glanz@rockets.utoledo.edu (A.G.); Sukanya.Chakravarty@rockets.utoledo.edu (S.C.); merina.varghese@rockets.utoledo.edu (M.V.); anita.kottapalli@rockets.utoledo.edu (A.K.); shumin.fan@rockets.utoledo.edu (S.F.); 2 Department of Physiology and Pharmacology, University of Toledo College of Medicine and Life Sciences, Toledo, OH 43614, USA; ritu.chakravarti@utoledo.edu

**Keywords:** IRF3, interferon, posttranslational modifications, RIPA, innate antiviral immunity, viral antagonism, SARS-CoV-2

## Abstract

The immune system defends against invading pathogens through the rapid activation of innate immune signaling pathways. Interferon regulatory factor 3 (IRF3) is a key transcription factor activated in response to virus infection and is largely responsible for establishing an antiviral state in the infected host. Studies in *Irf3^−/−^* mice have demonstrated the absence of IRF3 imparts a high degree of susceptibility to a wide range of viral infections. Virus infection causes the activation of IRF3 to transcribe type-I interferon (e.g., IFNβ), which is responsible for inducing the interferon-stimulated genes (ISGs), which act at specific stages to limit virus replication. In addition to its transcriptional function, IRF3 is also activated to trigger apoptosis of virus-infected cells, as a mechanism to restrict virus spread within the host, in a pathway called RIG-I-like receptor-induced IRF3 mediated pathway of apoptosis (RIPA). These dual functions of IRF3 work in concert to mediate protective immunity against virus infection. These two pathways are activated differentially by the posttranslational modifications (PTMs) of IRF3. Moreover, PTMs regulate not only IRF3 activation and function, but also protein stability. Consequently, many viruses utilize viral proteins or hijack cellular enzymes to inhibit IRF3 functions. This review will describe the PTMs that regulate IRF3′s RIPA and transcriptional activities and use coronavirus as a model virus capable of antagonizing IRF3-mediated innate immune responses. A thorough understanding of the cellular control of IRF3 and the mechanisms that viruses use to subvert this system is critical for developing novel therapies for virus-induced pathologies.

## 1. Introduction

### 1.1. IRF3, a Member of the Family of Interferon Regulatory Factors (IRFs)

IRF3 belongs to the interferon regulatory factor (IRF) family of transcription factors, which consists of nine members in mammalian cells [1,2]. IRFs have diverse cellular roles, including innate immunity, cell cycle progression, apoptosis, and tumor suppression [2,3]. These distinct functions are conferred by the activity of specific domains of the protein that allow it to form homo and heterodimers, which interact with other transcription factors or possess intrinsic transactivation potential [4,5]. Furthermore, the cell-type-specific expression of IRFs determines their respective function within a particular tissue [4]. Among the IRF family members, IRF3 and IRF7 are key regulators of innate antiviral immunity via the induction of type-I interferons (IFN) [6]. Upon microbial infection, the cellular innate immune sensors, the pattern recognition receptors (PRRs), located on the membranes or in the cytosol, recognize the microbial components known as pathogen-associated molecular patterns (PAMPs) [7]. The PRRs, upon binding to the PAMPs, relay the signal to downstream adaptors, leading to the formation of protein complexes, which activate transcription factors, e.g., IRF3, to induce the transcription of antiviral genes, such as type-I IFNs, e.g., IFNβ [8]. In addition to inducing antiviral genes, IRF3 also mediates innate immunity against virus infection by activating an antiviral, pro-apoptotic pathway [9]. IRF3 functions are regulated by posttranslational modifications (PTMs), e.g., phosphorylation, ubiquitination, and others, which alter protein conformation to relieve autoinhibition or allow interactions with additional binding partners. Such regulation is tightly controlled to avoid excessive inflammation and maintain cellular immune homeostasis. Therefore, this review will focus on the numerous PTMs that regulate IRF3 functions in the virus-infected cells. Finally, we describe how coronaviruses, including the SARS-CoV-2, antagonize the activation of IRF3.

### 1.2. IRF3: Protein Expression and Structure

Unlike other IRFs, which express cell-type-specifically or induced upon stimulations, IRF3 is expressed ubiquitously in most cell types and tissues, regardless of activation status [4,5]. The ubiquitous expression emphasizes the critical role IRF3 plays in innate antiviral immunity. As protein structure determines function, it is critical to understand the domain structure of IRF3 and how particular regions of the protein confer unique functional activities. As a transcription factor, IRF3 contains an N-terminal DNA-binding domain (DBD), whose homology is shared with other IRFs [4]. The DBD contains three alpha-helices, a four-stranded β-sheet, and loops that bind a GAAA consensus motif within the IFN-stimulated response elements (ISRE) of target genes [10]. Near the C-terminal end, IRF3 possesses an IRF association domain (IAD), which is responsible for its homo- and hetero-dimerization capabilities, with another monomer of IRF3 or another IRF, respectively [4]. At the C-terminus, the presence of an autoinhibitory domain forms a hydrophobic core to prevent dimerization and thus retain the protein in the cytosol at a resting state [11,12]. The autoinhibitory domain is also shared with IRF7, in which IRF3 has the highest degree of homology [12]; this similarity is unsurprising, given the antiviral role of IRF7 in immune cell subsets, such as dendritic cells. IRF3 remains inactive in the cytosol of uninfected cells; upon stimulation of PRRs by viral nucleic acids, key residues located in the signal response domain (RD) are phosphorylated by IKK-related kinases to allow conformational changes that promote dimerization through the now exposed IAD and nuclear translocation [4]. Thus, protein activity is highly dynamic, and mutation or posttranslational modification of specific residues within each of these domains can abolish one function without impacting a separate function of the protein. In all, the endogenous IRF3 protein is 427 amino acids in length [4,5]. Several IRFs also exhibit splice variants, including IRF3, whose DBD is truncated by differential splicing upon human papilloma virus (HPV) infection [13].

## 2. Role of IRF3 as a Mediator of Innate Immune Responses to Viral Infection

### 2.1. PRRs and Associated Pathways That Lead to IRF3 Activation

**Figure 1:**
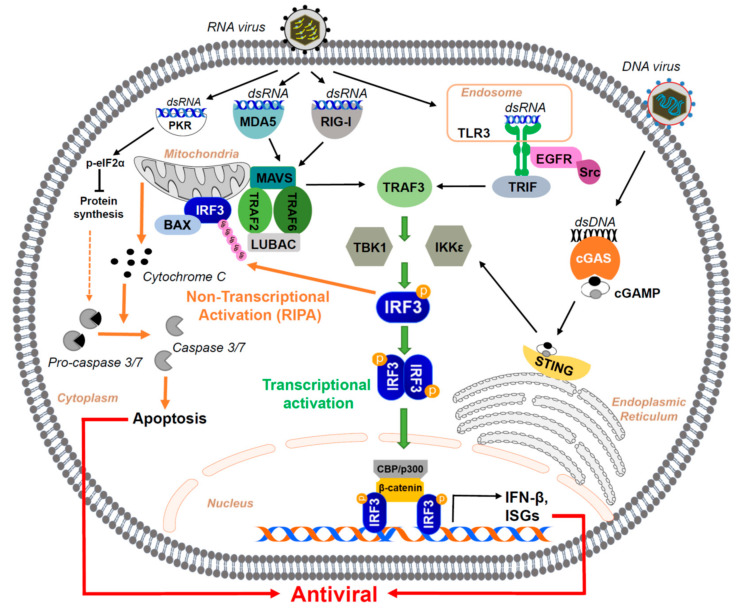
The transcriptional and non-transcriptional activation of IRF3 by virus-activated signaling pathways lead to host antiviral response. The PRRs, e.g., TLR3, RIG-I, MDA-5, and cGAS, present at different cellular locations, recognize the viral nucleic acids (RNA or DNA) upon their entry into the host cell. In the transcriptional activation of IRF3, the PRR-mediated signaling pathways activate the downstream kinases, TBK1 and IKKε, and directly phosphorylate the cytosolic IRF3. The phosphorylated IRF3 translocates to the nucleus to transcribe the antiviral genes, e.g., IFNβ and ISG. In the non-transcriptional pathway, IRF3 gets activated by ubiquitination and translocates to the mitochondria to activate the intrinsic caspases, leading to apoptotic cell death. The transcriptional and non-transcriptional pathways of IRF3 contribute to the overall antiviral response of the host. A dsRNA-binding protein PKR can also trigger cellular apoptosis by activating eIF2α, which inhibits protein synthesis.

The PRRs are transmembrane or cytoplasmic proteins that bind to non-self components, e.g., the PAMPs, to activate the downstream intracellular signaling cascades (Figure 1) [7,14]. Some of the PRRs also recognize the “danger signals” released from damaged cells, known as danger-associated molecular patterns (DAMPs), that indicate changes in homeostasis in the cell [7,14]. Among the PRRs, the Toll-like receptors (TLRs) and C-type lectin receptors (CLRs) are transmembrane, whereas the retinoic acid-inducible gene (RIG)-I-like receptors (RLRs), cyclic guanosine-monophosphate adenosine-monophosphate synthase (cGAS), and NOD-like receptors (NLRs) are cytoplasmic [14,15,16].

TLRs are type-I transmembrane proteins that contain ectodomains comprised of leucine-rich repeats (LRRs), which recognize the PAMPs or DAMPs [17]. TLRs also contain a Toll-interleukin 1 (IL1) receptor (TIR) domain that the adaptor proteins bind to, thus activating downstream signaling pathways [7,17]. Non-self RNAs such as viral double-stranded RNA (dsRNA), or self-RNAs such as small interfering RNA (siRNA) or those produced by damaged cells, serve as PAMPs and DAMPs for TLR3 [18]. Bacterial lipopolysaccharide (LPS) is recognized by TLR4, while TLR5 recognizes bacterial flagellin [18]. C-type lectin receptors are the second class of transmembrane PRRs that also play a vital role in recognizing the viral PAMPs. Cells expressing CLRs mainly include myeloid cells such as monocytes, macrophages, and dendritic cells [19,20]. They have long been known for their role in anti-fungal immunity; however, studies have demonstrated their function in virus (such as HIV-1) recognition, leading to their lysosomal degradation or transmission [19,21,22]. Among the cytoplasmic RNA sensors, RIG-I and melanoma differentiation-associated gene 5 (MDA5) recognize the viral RNA. These receptors contain tandem domains on their N-terminus: a caspase activation and recruitment domain (CARD) and a DExD/H-box domain. Upon RLR stimulation by viral RNA, the CARD domains are released, leading to aggregation of the K63 polyubiquitin chains to CARD tetramers that bind to the MAVS adaptor protein to activate downstream signaling [23,24,25,26]. The RLRs identify features that are common to both viral genomes and replication intermediates, such as 5′ triphosphate RNA, long dsRNA, etc., to distinguish between foreign and endogenous RNA [27]. The 5′-capping of the endogenous mRNA protects them from being recognized by RLRs [28,29].

The RLRs use MAVS (also known as IFNβ promoter stimulator 1 (IPS-1), VISA, CARDIF) as their adaptor protein [30,31,32,33]. TLR3 signals through the recruitment of its adaptor TRIF, a receptor-specific adaptor molecule, whereas other TLRs use MyD88 for signaling [30]. Recruitment of the adaptor proteins by TLR3 or RLR results in the activation of two non-canonical IκB kinases: TBK1 (TANK-binding kinase 1) and IKKε to the adaptor protein (TRIF/MAVS/STING). These kinases directly catalyze the phosphorylation of IRF3, resulting in its transcriptional activation [34,35]. The cGAS-STING pathway plays a major role in antiviral IFN-β production on recognizing pathogenic dsDNA. The cGAS changes conformation to result in the production of cyclic GMP-AMP (cGAMP) that interacts with the endoplasmic reticulum-resident protein stimulator of Interferon genes (STING) [15,16,36]. The activated STING interacts with TBK1/IKKε to phosphorylate IRF3 and result in IFN-β production. Similar to IRF3, the TLR3, RLR, and cGAS signaling pathways also activate additional transcription factors, e.g., NF-κB and other IRFs, e.g., IRF7 [6,14]. A recent study indicated that additional phosphorylation sites on IRF7 are required for transcriptional activity in response to STING and MAVS signaling [37].

### 2.2. Transcriptional Activation of IRF3: Induction of IFN and Antiviral Genes

The recognition of viral PAMPs by the cellular PRRs, via intracellular signaling cascades, activates IRF3 for its transcriptional function (Figure 1). The adaptor proteins, such as MAVS, STING, and TRIF, are phosphorylated at a conserved *p*L*x*IS motif by kinases, which results in the binding of IRF3 to these adaptor proteins [38]. This binding brings IRF3 in close proximity with the kinases such as TBK1 and IKKε that are responsible for IRF3 phosphorylation, which results in its dimerization [34,35]. Activated, dimerized IRF3 translocates to the nucleus to bind to the promoters of the target genes, e.g., the *IFNB1*. On the gene promoter, the activated IRF3 recruits a complex containing the transcriptional coactivator proteins, e.g., CREB-binding protein (CBP) and p300 [39,40]. We have shown that the activated IRF3 requires the deacetylated β-catenin to recruit the transcriptional coactivators [41]. The deacetylation of β-catenin is mediated by the cytoplasmic deacetylase, HDAC6 [41,42]. This complex with IRF3 is indispensable for the DNA binding activity of IRF3 as a transcription factor [40,43]. The binding of the CBP/p300-IRF3 complex to the DNA is mediated by a Q-rich domain present on p300, and its histone acetyltransferase (HAT) activity is an important requirement for this function [43]. The binding of this complex results in the initiation of IFNβ gene transcription. IFNβ is a secreted cytokine, which binds to its receptor IFNAR (IFN-α/β receptor) expressed on the surface of the infected or yet uninfected cells [24,44,45]. As a result, IFN signaling through the JAK/STAT pathway activates ISGF3, which induces IFN-stimulated genes (ISGs), which help establish an antiviral state in the infected cell by restricting specific stages of the virus replication [24,44]. Many of these ISGs are induced directly by IRF3, without the requirement of IFN signaling [46].

### 2.3. Non-Transcriptional Activation of IRF—The Pro-Apoptotic Pathway

**Figure 2:**
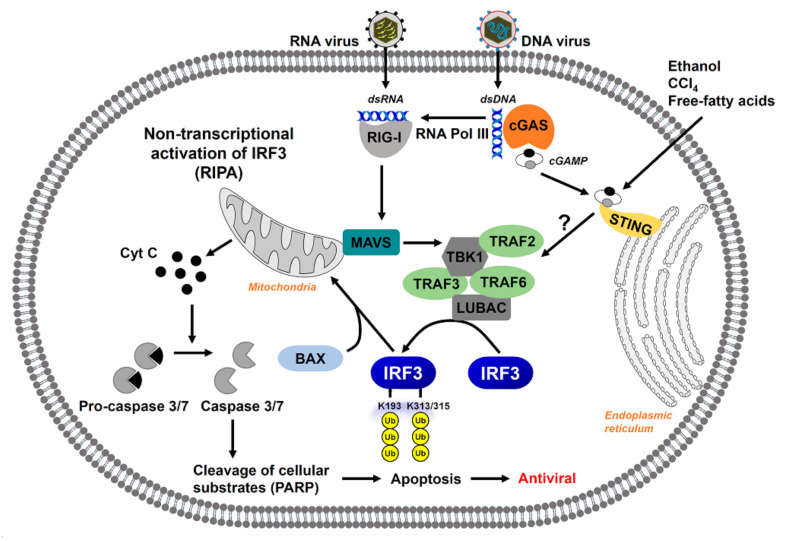
The non-transcriptional activation of IRF3 by RIPA causes cell death. IRF3 can be activated by RNA or DNA virus infection, which triggers RIG-I or cGAS signaling pathways. The RIG-I activation by RNA or DNA viruses can recruit a complex by TBK1/TRAF2/TRAF6, which allows the binding of IRF3 to LUBAC. The LUBAC-mediated linear ubiquitination of IRF3 leads to its translocation to the mitochondria and interaction with BAX. Mitochondrial translocation of IRF3/BAX complex leads to cytochrome C release that activates the apoptotic caspases, leading to apoptotic cell death. This pathway has been named ‘RIPA’, which can also be triggered by ethanol, CCL4, and free fatty acids, which activate the PRR signaling pathways.

In addition to its role in the transcription of antiviral genes, IRF3 has a transcription-independent function in triggering apoptosis of virus-infected cells (Figure 2). We have uncovered that IRF3, activated by the RLR signaling pathway, triggers apoptotic cell death through the RLR-induced IRF3-mediated pathway of apoptosis (RIPA) [9]. RIPA can be induced by the infection with RNA viruses, including Sendai virus (SeV), encephalomyocarditis virus (EMCV), or vesicular stomatitis virus (VSV), or transfection of dsRNA, which is recognized by the RIG-I like receptors [47,48,49,50]. RIPA can also be induced by the DNA virus (e.g., adenovirus) infection or poly(dA:dT) transfection, which activates RIG-I via RNA Pol III [51]. In contrast to transcriptional activation, TLR stimulation fails to activate IRF3′s pro-apoptotic response, indicating RIPA’s specificity [49]. Thus, the pro-apoptotic pathway is distinct from IRF3′s transcriptional pathway and is regulated independently. Although the signaling pathways that result in the two outcomes of IRF3 activation are discrete, they do share some common features, among them the proteins RIG-I, MAVS, TRAF3, and TBK1 (Figure 2) [49]. Alternatively, TRAF2, TRAF6, and LUBAC were found to be dispensable for transcription but required for RIPA [48]. Activation of IRF3 in RIPA requires its ubiquitination by LUBAC on two key lysine residues: K193 and K313/315 [48]. This modification enables IRF3 to associate with the pro-apoptotic protein Bax through a BH3 domain and translocate to the mitochondria to carry out the intrinsic apoptotic cascade [49].

As a process that specifically targets virus-infected cells for elimination, one might expect that RIPA benefits the host as an antiviral pathway. Early studies of RIPA uncovered an antiviral role for IRF3-mediated apoptosis by preventing viral persistence in SeV and hPIV3-infected cells [50]. Moreover, an in-depth investigation of the interplay between RIPA activation and SeV replication revealed a deficiency in any fundamental component of the apoptotic pathway predisposed cells to persistent infection [47,50]. In order to determine the relative contribution of RIPA to prevent viral pathogenesis, SeV infection was studied in knock-in mice expressing the S1 mutant of Irf3, which is RIPA-active but defective in the transcriptional pathway [48]. In the absence of IRF3-mediated gene transcription, the *Irf3^S1/S1^* mice were protected from lethal infection with SeV, demonstrating the importance of RIPA in protection from respiratory viral pathogenesis. Of note, apoptosis of virus-infected cells can benefit the host or the virus, depending on circumstance. SeV temporally regulates RIPA by suppressing apoptosis early during infection while later relieving the inhibition to rapidly kill the infected cell by the host to clear virus infection [50,52]. Cytomegalovirus uses viral proteins to block the activation of Bax, thereby inhibiting apoptosis [53]. These studies indicate apoptosis may be a common target for virus antagonism of the host antiviral response.

In addition to RIPA, IRF3 participates in RIPA-like pathways activated by STING, both in viral and non-viral contexts. A study on human T cell leukemia virus type 1 (HTLV-1) demonstrated the virus activates an apoptotic pathway in primary human monocytes [54]. Mechanistically, HTLV-1-induced monocyte apoptosis occurs through the STING-driven IRF3-Bax complex, similar to RIPA. Beyond its antiviral function, IRF3-mediated apoptosis has been implicated in mitotic cell death of non-small cell lung carcinoma cells; consequently, IRF3 expression sensitized cells to the anti-mitotic agent Taxol [55]. Additional non-viral triggers have been shown to induce the STING/IRF3/Bax apoptotic pathway, including ethanol, CCl_4_, and free fatty acids [56,57,58]. All three of these inducers contribute to liver injury, implicating IRF3 in the development of liver disease pathology. Studies through our work revealed that RIPA in restorative hepatic monocytes contributes to ethanol-induced liver injury in an acute-on-chronic hepatitis model [59]. In contrast, we further showed that the non-transcriptional RIPA activity of IRF3 plays a protective role in high-fat diet-induced liver injury [59]. Therefore, while we have shown the activation of IRF3-driven apoptosis in virus infections benefits the host, the role of IRF3 in liver disease still remains somewhat unclear. Consequently, this will be a worthwhile area of research in the future.

### 2.4. Regulation of Non-Transcriptional Function of IRF3

Because RIPA contributes to the antiviral activity of IRF3, it was thought that pharmacological activation of RIPA might be beneficial to the host. To address this, we performed a high throughput screening of a library of FDA-approved compounds for their ability to promote RIPA. The screen isolated a small subset of compounds that promoted the RIPA function of IRF3 in human and mouse cells [60]. Doxorubicin, a known anticancer drug, was found to be a strong RIPA-activating agent. The RIPA-activating function of doxorubicin was dependent on the ERK signaling pathway. Doxorubicin was found to be antiviral against VSV, herpes simplex virus (HSV-1), and the antiviral activity depends on the RIPA function of IRF3. The hypothesis that small molecules can activate RIPA to exhibit their antiviral activity was further validated using pyrvinium pamoate, another RIPA-activating compound. Pyrvinium pamoate promoted RIPA via ERK signaling pathway and is antiviral against VSV and HSV-1. Overall, our study is a strong foundation for future research to identify molecules that trigger RIPA in both viral and non-viral contexts to exhibit therapeutic activities. RIPA, triggered by SeV infection, is temporally regulated by PI3 kinase-mediated activation of AKT to inhibit the early induction of apoptosis. The virus-activated PI3K/AKT inhibits the degradation of XIAP, an inhibition of apoptosis. Later in the infection, IRF3/BAX-mediated activation of intrinsic apoptotic pathway releases the PI3K/AKT-mediated inhibition of RIPA [52]. Therefore, pharmacological regulators of PI3K and AKT pathways can be used to regulate RIPA. Endogenous RIPA regulators have already been identified, highlighted by a recent study that found the p150 isoform of the RNA-editing enzyme ADAR1 prevents sustained RIG-I activation during influenza virus infection [61]. Interestingly, a role for IRF3-mediated apoptosis has also been described in the study of liver diseases, suggesting the physiological role of RIPA may extend beyond virus infection [56,62].

## 3. Regulation of IRF3 Functions by Posttranslational Modifications

### 3.1. IRF3 in Uninfected Cells

The subcellular localization of proteins controls their functions, and IRF3 is no exception. Many transcription factors, including IRFs, NF-κB, and STATs, remain in the cytoplasm prior to activation [63]. This may be a mechanism to maintain homeostasis by preventing excessive production of downstream genes in the absence of a stimulus. IRF3 contains both a nuclear export signal (NES) and a nuclear localization signal (NLS) that allow it to shuttle between the nucleus and cytoplasm [63]. However, the export of IRF3 is dominant, resulting in a greater pool of IRF3 localized to the cytoplasm in the absence of infection. The crystal structure of IRF3 revealed the presence of autoinhibitory domains that keep IRF3 in an inactive conformation at a resting state [12]. Hydrophobic residues located within the autoinhibitory domains restrict IRF3 dimerization in the absence of a stimulus [12]. Phosphorylation of IRF3 induced by virus infection triggers a rearrangement of the helices responsible for autoinhibition, restructuring IRF3 to allow dimerization and expose the DNA-binding domain [12]. A study, before the crystal structure of IRF3 was available, described the involvement of the C-terminal region located between amino acids 380–427 and an N-terminal region between amino acids 98–240 in IRF3 autoinhibition [64].

### 3.2. Phosphorylation of IRF3

#### 3.2.1. Phosphorylation-Mediated Activation of IRF3

IRF3 activation involves the virus-induced recruitment of two non-canonical IκB kinases, TBK1 (TANK-binding kinase-1) and IKKε (IKB kinase-ε), catalyzing an important conformational change required for dimerization and translocation to the nucleus [4]. The autoinhibitory domain at the IRF3 C-terminal domain contains amino acids 380–427, which, along with the helices at the N-terminal domain, form a compact structure blocking activation [12,65]. This structure results in the masking of certain hydrophobic residues on the IAD, which include Leu^322^, Pro^324^, Ile^326^, Val^327^, Leu^329^, Ile^330^, Cys^371^, Ala^374^, Leu^375^, Met^378^, and Ala^379^ distributed in the two helices (H3 and H4) that are present in this domain [11,12]. Phosphorylation results in exposure of the IAD, which allows its association with another IRF3 or IRF7 monomer to form a homo- or heterodimer, respectively [12].

**Figure 3:**
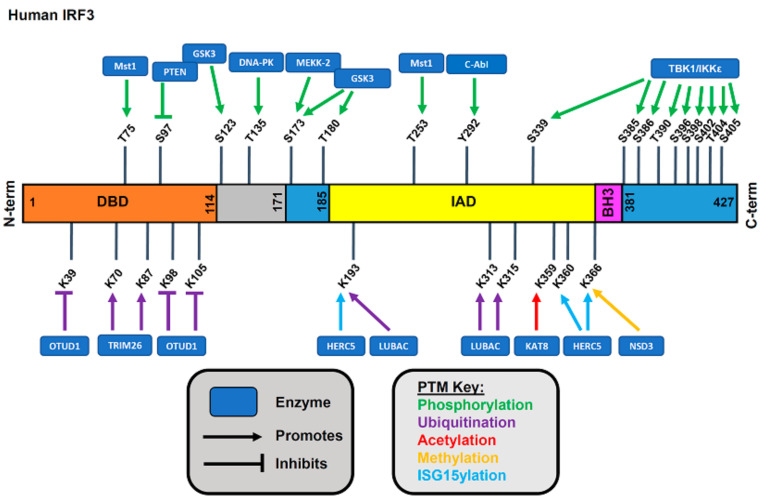
Activation of IRF3 by posttranslational modifications. Human IRF3 and its various functional domains are shown; DBD, DNA-binding domain, IAD, IRF association domain, BH3, and Bcl2 homology domain. The amino acids that are posttranslationally modified are indicated, and their respective enzymes catalyze these modifications.

Phosphorylation occurs at two clusters containing seven total critical sites within its C-terminal domain (Figure 3). Phosphorylation at cluster 1, including Ser^385^ and Ser^386^, is widely accepted as essential for IRF3 dimerization [4]. Phosphorylation at cluster 2, including sites Ser^396^, Ser^398^, Ser^402^, Thr^404^, and Ser^405^, relieves autoinhibition and allows binding of the coactivator CBP to facilitate further phosphorylation at site 1 [4]. Taken together, a dual model of activation occurs among the two clusters, while no consensus on the individual roles of the C-terminal phospho-acceptor sites has been reached [66]. Among these sites, phosphorylation at Ser^386^ and Ser^396^ is critical for IRF3 activation [65]. Lastly, phospho-acceptor sites Thr^390^ and Ser^339^ modulate the IRF3 activity. While not initially implicated in early reported IRF3 activation, Thr^390^ has been identified as a novel in vivo phosphorylation target that functions to promote phosphorylation at the critical TBK1-targeted site Ser^396^ [67]. Phosphorylation at Ser^339^ is reported to play a role in achieving optimal IRF3 transactivation capacity that has been undetected by conventional methods due to lack of sensitivity [68]. Various cellular conditions involve phosphorylation-mediated upregulation of IRF3 activity. DNA-dependent protein kinase (DNA-PK) binds and phosphorylates IRF3 at Thr^135^, extending its half-life with reported greater nuclear retention and delayed degradation by the ubiquitin-proteasome pathway [69]. The nonreceptor tyrosine kinase c-Abl, primarily performing its role in cellular and humoral immunity, phosphorylates IRF3 at site Tyr^292^ to promote IRF3 activation achieved primarily through TBK1-mediated phosphorylation at Ser^396^ [70]. This involvement in innate immunity offers complexity to the mechanism by which loss of c-Abl kinase results in viral susceptibility [70]. A unique example of phosphatase rather than kinase-mediated upregulation of IRF3 activity is demonstrated by the critical tumor-suppressor phosphatase and tensin homolog (PTEN) and its activity at IRF3 site Ser^97^, ultimately driving IRF3 import into the nucleus [71].

#### 3.2.2. Phosphorylation-Mediated Downregulation of IRF3 Activity

The phosphorylation may also result in attenuated antiviral signaling, primarily through either blocked homodimerization or enhanced degradation of IRF3. For instance, mammalian sterile 20-like kinase Mst1 functions as a negative regulator of IRF3 by blocking two key components of the IRF3 activation pathway, namely promoter binding and homodimerization. Direct phosphorylation at Thr^75^ located within the DBD of IRF3 impedes its ability to bind chromatin, presumably due to interference with its proximal Arg^78^ capacity to form hydrogen bonds to nucleotides, while phosphorylation at Thr^253^ disrupts the critical IRF3 homodimerization likely by electrostatic repulsion and steric hindrance [72]. As a regulator of various cellular processes, Mst1 may link cellular stress with appropriate levels of antiviral response. Another example resulting in downregulated IRF3 activity is the Glycogen synthase kinase 3 (GSK3)-mediated phosphorylation of IRF3 at its proline-rich linker region including Ser^123^, Ser^173^, and Thr^180^ residues and the nuclear export signal, ultimately impeding strong binding of target DNA by decreased flexibility [73]. The activity of GSK3 occurs in a manner similar to the Epstein Barr Virus BGL4 kinase-mediated suppression of IRF3 at Ser^123^, Ser^173^, and Thr^180^ in which decreased DNA binding ability was observed as part of a conserved viral mechanism evolved to downregulate IRF3 signaling effects [74]. Enhanced degradation of IRF3 is a mechanism by which the antiviral response is inhibited. An example of this is phosphorylation-dependent polyubiquitination by the peptidyl-prolyl isomerase Pin1, found to have increased expression in malignancy. Double-stranded RNA- induced phosphorylation of the Ser^339^-Pro^340^ motif is recognized by peptidyl-prolyl isomerase Pin1 through its Trp residues, catalyzing a conformational change and ultimately leading to IRF3 polyubiquitination and proteasome-dependent proteolysis [75]. Another mechanism by which conditions of malignancy diminish innate immunity involves MEKK2, a kinase in macrophages involved in the process by which cancer cells transform the microenvironment. MEKK2-mediated disruption of innate antiviral immunity occurs by its direct phosphorylation of IRF3 at Ser^173^, triggering IRF3 polyubiquitination, specifically through upregulating Lys^33^ (K33)-linked polyubiquitination on the NLS motif in a non-degradative fashion [76]. The roles of both Pin1 and MEKK2 in their unique suppression of the IRF3 pathway demonstrate possible mechanisms by which cancer cells promote greater susceptibility to viral infection. This is similarly demonstrated in the mechanism by which mutated PTEN is unable to employ the dephosphorylation-induced IRF3 activation of its wildtype counterpart.

### 3.3. Ubiquitination of IRF3

#### 3.3.1. Protein Ubiquitination

Protein ubiquitination, like phosphorylation, is a reversible process that regulates protein functions [77]. Ubiquitination is catalyzed by a series of enzymes called E1, E2, and E3, which sequentially activate, conjugate, and ligate a molecule of ubiquitin (Ub) onto a target protein [77]. Ubiquitin itself is a small polypeptide, 76 amino acids in length, which is attached to a target protein through the formation of an isopeptide bond between its C-terminal glycine and the epsilon amino acid residue of the lysine within the target protein. There are additional internal lysine residues within ubiquitin that can be linked to other ubiquitin molecules to form a chain. The lysine residue used for chain formation determines the functional outcome of the ubiquitination of the target protein. For instance, K48-linked ubiquitin chains typically drive proteasomal degradation of the target, while K63-linked chains are known for modulating the protein activity and enhancing protein–protein interactions to facilitate signal transduction [78]. There are also other, relatively less common ubiquitin linkages: M1, K0, K6, K11, K27, K29, or K33 [79]. The M1 linkage involves linkage to the amino-terminal methionine of the next ubiquitin molecule, and therefore this type of linkage is also referred to as “linear ubiquitination”; currently, the only known E3 ligase capable of linear ubiquitination is the linear ubiquitin chain assembly complex (LUBAC) [80]. In addition to these chains, a single moiety of ubiquitin can be conjugated to the target protein resulting in monoubiquitinated protein [81,82]. As a reversible posttranslational modification, ubiquitin chains can be hydrolyzed by de-ubiquitinating enzymes (DUBs) to remove the ubiquitin chain [83]. Over 600 E3 ligases and 90 DUBs are encoded by the human genome, indicating the importance and specificity of this process [77,83]. Moreover, ubiquitin-mediated signaling does not act in isolation, but rather participates in the crosstalk between different posttranslational modifications that work together to carefully control activation and inactivation of signaling cascades. Ubiquitin has a critical role in modulating signaling activation from all three classes of pattern recognition receptors (PRRs): Toll-like receptors (TLRs), RIG-I-like receptors (RLRs), and NOD-like receptors (NLRs) [77].

#### 3.3.2. Ubiquitin-Mediated Degradation of IRF3

While IRF3 activation is key to an optimal antiviral response, excessive activity of IRF3 can be detrimental to the host. As such, the ubiquitination of IRF3 that targets the protein for destruction is a negative regulatory mechanism by which the body returns to homeostasis after active infection. Moreover, many viruses trigger IRF3 degradation, which helps facilitate viral replication [84,85]. The K48-linked ubiquitination is the Ub-linkage commonly associated with proteasomal degradation. A large number of cellular ubiquitin ligases carry out this function; the high number of E3 ligases available ensures specificity in the protein substrates targeted for degradation.

One of the first reports of direct IRF3 ubiquitination mediating IRF3 degradation describes a role for Cullin1-based E3 ligases [86]. The RING family is the largest among the groups of E3 ligases, characterized by multi-protein complexes containing three invariable subunits, including Cullin1. Together, the complex is named Skp, Cullin, F-box containing complex (SCF), after its components. Following Sendai virus infection, phosphorylation of IRF3 allows for efficient recognition by an endogenous SCF complex, which then induces polyubiquitination to target IRF3 for the proteasome. This process helps regulate the steady-state levels of IRF3 to finetune IRF3 activation during virus infection.

In contrast to Pin1 and the unidentified Cullin 1-based ubiquitin ligase, which only targets activated IRF3 for degradation, the ubiquitin ligase RAUL targets both resting and activated IRF3 to regulate type I IFN levels [87]. The binding between RAUL and IRF3 is independent of its phosphorylation state, as IRF3 phosphorylation mutants retained their ability to bind RAUL. The direct interaction between the two proteins occurs through the N-terminal regions of IRF3 (residues 1–128) and RAUL (residues 1–655). Both the substrate-binding domain and the catalytic domain of RAUL were required for proteolysis of IRF3, and the catalytically inactive RAUL mutant functioned in a dominant-negative fashion to inhibit RAUL-dependent IRF3 ubiquitination. Importantly, both basal levels and virus-stimulated increases in IFN-production were suppressed by RAUL activity, resulting in enhanced viral replication in cells ectopically expressing RAUL. In all, these data support a role for RAUL as a ubiquitin ligase targeting IRF3 for proteasomal degradation through K48-linked ubiquitination.

Another ubiquitin ligase that negatively regulates type I IFN production during viral infections by modification of IRF3 is the RBCC protein interacting with PKC1 (RBCK1) [88]. Like RAUL, overexpression of RBCK1 caused a reduction in IRF3 protein levels, which was rescued by MG132, the proteasomal inhibitor. The N-terminal UBL domain (1–220) of RBCK1 was required for interaction with IRF3. Not long after the role of RBCK1-mediated IRF3 ubiquitination was discovered, several additional ubiquitin ligases targeting IRF3 were identified. Ro52 (TRIM21) also regulates the activity of the IFNβ promoter downstream of TLR4, TLR3, and RIG-I activation [89]. Interaction with IRF3 depends upon the C-terminal SPRY domain of Ro52, previously reported to determine the substrate specificity of the TRIM family of proteins [90,91]. The endogenous interaction between Ro52 and IRF3 was dependent upon stimulation; no association was observed at a resting state [89]. In addition to Ro52, TRIM26, another member of the tripartite motif (TRIM) family has been reported to directly target IRF3 for degradation [92]. TRIM26 predominately promotes K48-linked polyubiquitination of IRF3 at the residues K70/K87 to promote proteolysis. The ubiquitin ligase was found to interact with IRF3 in the nucleus following TLR4 activation and SeV infection in macrophages. This evidence suggests activated IRF3 is the target for TRIM26-mediated ubiquitination. Additionally, the E3 ubiquitin ligase RING finger protein 26 (RNF26) stimulates the autophagic degradation of IRF3 upon overexpression [93]. Moreover, RNF26 catalyzes the polyubiquitination of IRF3′s upstream adaptor STING, which responds to cytosolic DNA-induced type I IFN. However, a direct role for RNF26 in binding or ubiquitinating IRF3 was not observed.

A study by Lei et al. revealed a role for the transcription factor Forkhead box protein O1 (FoxO1) as a negative regulator of virus-induced IFNβ production [94]. FoxO1 had previously been reported to interfere with innate immune activation by inhibition of IRF7 transcriptional activity [95]. In addition, FoxO1 was also found to interact with IRF3 in the cytosol of virus-infected cells and trigger K48-linked polyubiquitination of IRF3, promoting its proteasomal degradation [94]. While virus infection was required for FoxO1-mediated negative regulation of IRF3, the mRNA level of FoxO1 remained unchanged post-infection, suggesting posttranslational control of FoxO1 activity. Interestingly, a knockdown of neither RBCK1 nor RAUL affected FoxO1-dependent IRF3 degradation, indicating FoxO1 may degrade IRF3 via an unknown E3 ligase. This study suggests FoxO1 as a novel target for therapeutic intervention of viral diseases or IRF3-driven pathology.

The protein casitas B-lineage lymphoma (c-Cbl) is a recently identified novel ubiquitin E3 ligase targeting IRF3 [96]. The c-Cbl is a negative regulator of receptor tyrosine kinases, and other studies have reported its role in regulating innate immunity through suppression of RIG-I and IRF8 [97]. While c-Cbl interacts directly with IRF3 under normal physiological conditions, VSV infection enhances the interaction between the two proteins. The IAD of IRF3 and the tyrosine kinase binding (TKB) domain of c-Cbl are required for the interaction. The c-Cbl mediates K48-linked polyubiquitination of IRF3 to promote its degradation and downregulate IFNβ production to negatively regulate the antiviral response. IRF3 is also degraded via proteolytic cleavage by caspase-8, activated in virus-infected cells [84]. The caspase cleavage of IRF3 promotes its ubiquitination and proteasomal degradation. The mutation of the caspase cleavage site inhibits IRF3 degradation.

#### 3.3.3. Ubiquitin-Mediated Functional Change in IRF3 Activity

In addition to promoting degradation of IRF3, ubiquitination can modulate IRF3 activity, thus regulating the antiviral response. A study by Zhang et al. reports non-canonical K6-linked ubiquitination of IRF3 is indispensable for the binding of IRF3 to gene promoters [79]. The ubiquitin-conjugating enzyme Ubc5 was found to be required for IRF3 activation [98]. Mutation of the Ubc5 catalytic site abrogated dimerization of IRF3, demonstrating a role for this enzyme in virus-induced IRF3 activation [98]. In a model of autoimmune disease, K63-linked ubiquitination of IRF3 on Lys98 activated IRF3 for its transcriptional function [99]. However, ubiquitination can also suppress IRF3 activity, as K33-linked ubiquitin chains on Lys77 inhibit IRF3 nuclear translocation [76]. This modification prevents the IRF3 association with the importin required for its entry into the nucleus. We have shown that a linear ubiquitination-mediated posttranslational modification of IRF3 triggers the virus-induced apoptotic pathway by RIPA [48]. Therefore, specific ubiquitin linkage determines the functional outcomes of the targeted proteins.

#### 3.3.4. Deubiquitination of IRF3

Ubiquitination is a reversible process, and as such, there are deubiquitinase enzymes capable of removing the modification from IRF3. Namely, the ovarian tumor domain-containing deubiquitinase 1 (OTUD1) removes ubiquitin linkages from IRF3 and has been implicated in autoimmune disease as a result [99]. This enzyme directly interacts with IRF3 to catalyze the removal of K63-linked polyubiquitin chains on IRF3, thus suppressing IRF3 nuclear translocation and production of antiviral genes, including IFNβ [99]. Consequently, *OTUD1^−/−^* mice display an enhanced immune response against viral pathogens [99]. In addition to K63-linked ubiquitin, OTUD1 also cleaves K6-, K11-, and K29-linked ubiquitin chains from IRF3, although only K6-linkages were required for IRF3 binding to DNA [79]. Expression of OTUD1 was induced by a variety of stimuli, including VSV, herpes simplex virus 1 (HSV-1), SeV, polyI:C (TLR3), R848 (TLR7/8), and LPS (TLR4) [79]. Moreover, an increase in OTUD1 expression was observed upon IFNβ treatment, indicating OTUD1 may function as an ISG [79]. Collectively, these studies describe multiple roles for OTUD1 in the negative regulation of IRF3-mediated antiviral immunity.

### 3.4. SUMOylation

SUMOylation is a process similar to ubiquitination in that it uses the enzymes E1, E2, and E3 in a three-step process to conjugate SUMO, a small ubiquitin-like protein, to a specific lysine residue on the target protein [100,101]. It has been reported that virus infection triggers the SUMOylation of mouse Irf3 on Lys^152^, resulting in the downregulation of type I IFN production [102]. While Lys^152^ is not conserved in human IRF3, others have reported Lys^87^ of mouse Irf3 is also targeted for SUMOylation, and this residue is conserved in human IRF3 [103]. Under physiological conditions, the enzyme Sentrin/SUMO-specific protease (SENP) deSUMOylates IRF3, allowing for its ubiquitination and proteasome-mediated degradation [103]. Thus, in this scenario, crosstalk between SUMOylation and ubiquitination influences the activation and stability of IRF3. Interestingly, SUMO expression in cells infected by members of the *Rhabdoviridae* family of viruses can have differential effects, depending on the specific member of the virus family [104]. SUMO sensitizes cells to infection by rabies virus (RABV) by inducing the SUMOylation of IRF3, thereby preventing IRF3 activation and production of type I interferon [104]. However, SUMO expression protects against vesicular stomatitis virus (VSV) infection by blocking primary mRNA synthesis, thus inhibiting the production of viral proteins [104].

### 3.5. Glutathionylation

In addition to SUMOylation, a process called glutathionylation has also been reported for IRF3. Glutathione is a tripeptide composed of glutamate, cysteine, and glycine residues expressed at high levels in mammals, plants, yeast, and some bacteria [105]. Glutathionylation is a posttranslational modification in which glutathione is reversibly attached to the thiol group of a protein cysteine residue [106]. Glutathionylation serves as a regulatory mechanism of protein enzymatic activity and tightly regulates cell signaling pathways through the selective activation or inhibition of signaling proteins [107]. Essential roles for S-glutathionylation in inflammatory disease and viral infection have also been identified [105]. For instance, innate immune signaling pathways leading to the production of IFNβ are regulated by the activity of glutaredoxin 1 (GRX-1), a cytoplasmic enzyme responsible for deglutathionylation of target proteins [108]. The transcription factor IRF3 is glutathionylated in uninfected cells, but upon infection by the Sendai virus, GRX-1 mediates the deglutathionylation of IRF3 [108]. Deglutathionylation of IRF3 is required for its transcriptional function via recruitment and interaction with the coactivator CBP [108].

### 3.6. Methylation

There are several reports of direct methylation of IRF3 and other proteins involved in the pathway for IFNβ production. Methylation involves the transfer of a methyl group from the methyl donor S-adenosylmethionine (SAM) to a lysine or arginine residue on the target protein; this process is catalyzed by the enzymes called methyltransferases [109]. Although methylation is generally thought of as a process that regulates gene transcription, methylation has also been recently recognized as a regulator of other physiological processes, including the regulation of innate immune signaling [109,110]. Wang et al. identified a mechanism by which IRF3 becomes methylated by nuclear receptor-binding SET domain 3 (NSD3) upon viral infection [111]. Monomethylation of IRF3 occurs at Lys^366^ and is required for its transcriptional activity [111]. Additional studies of IRF3 methylation will be necessary to validate these findings and investigate the physiological relevance of IRF3 methylation in an animal model.

### 3.7. ISG15ylation

Interferon stimulated gene 15 (ISG15) is another ubiquitin-like modifier protein that is induced by type-I IFN [112]. As such, it plays an important role in regulating the antiviral response by its conjugation to the target proteins. ISG15 exhibits about 30% amino acid sequence homology with ubiquitin and contains two ubiquitin-like domains, revealed by cloning and crystal structure analyses [113,114,115]. Both viral proteins and cellular proteins have been identified as substrates for ISG15ylation thus far, although many of the presumed substrates are still yet to be identified [112]. ISG15 modifications participate in crosstalk with the ubiquitin and SUMO pathways, and many of the conjugating and deconjugating enzymes catalyze reactions for multiple types of Ub-like modifiers [116]. Moreover, some target proteins are common to both Ub and ISG15 conjugation pathways. For instance, UbcH8-mediated ISG15ylation of RIG-I suppresses ubiquitination by RNF125, thus protecting RIG-I from proteasomal degradation [117]. In addition to RIG-I, several other antiviral ISGs have been identified as ISG15 targets: PKR, MxA, and IFIT1 [118]. Furthermore, studies of ISG15-deficient mice have demonstrated ISG15 is critical for antiviral protection [112,116]. Therefore, it is unsurprising that IRFs are also targets of ISG15ylation. Type-I IFN induces expression of the ISG15 E3 ligase HECT domain and RLD 5 (HERC5) [119,120]. HERC5 catalyzes the addition of an ISG15 group onto IRF3 to sustain IRF3 activation by attenuating the interaction between IRF3 and Pin1 [121]. Cys^994^ of the HECT domain of HERC5 is required for its enzymatic activity, while the direct interaction between HERC5 and IRF3 requires amino acids 200–360 of IRF3. ISG15ylation occurs on residues Lys^193^, Lys^360^, and Lys^366^; mutation of these residues allows Pin1 to bind IRF3 tightly and negatively regulate type I IFN production. Therefore, this study by Shi et al. supports a positive regulatory role for the ISG15 E3 ligase HERC5 in augmenting innate immunity.

### 3.8. Acetylation

Acetylation is another posttranslational modification, which has been widely reported to regulate gene transcription by altering histone protein structure [122,123]. However, in the past couple of decades, more attention has been given to the role of acetylation in regulating the activity and stability of non-histone proteins. Enzymes called histone acetyltransferases (HATs) are responsible for the enzymatic addition of an acetyl group from a donor (acetyl coenzyme A) onto the ε amino group of lysine in the target protein [124]. Histone deacetylases (HDACs) remove the acetyl groups, and together these two enzymes create a balance between activation and inactivation of multiple host pathways [125]. In fact, the IFN signaling pathway is regulated by acetylation-dependent events [126]. All three components of the ISGF3 complex–STAT1, STAT2, and IRF9 are acetylated by CBP. Furthermore, several members of the IRF family are acetylated by HATs within their DNA-binding domain, which is highly conserved among IRFs and is critical for transcriptional activation [127]. Caillaud et al. were the first to map the residues of acetylation of an IRF family member–Lys^92^ of IRF7; previous studies reported acetylation of IRF1 and IRF2, but these studies lacked identification of the residues involved [124,128]. Suhara et al. discovered acetylation of the IRF3 holocomplex by p300 is required for DNA binding, although monomeric IRF3 was not a substrate for HAT activity [43]. We showed that the deacetylation of the IRF3 coactivator β-catenin by HDAC6 facilitates the formation of a stable transcription initiation complex in TLR/RLR signaling; however, acetylation of IRF3 itself was not observed [41]. Recently, an enzyme with ubiquitous expression in immune cells, called lysine acetyltransferase 8 (KAT8), was shown to catalyze acetylation of IRF3 [129]. Acetylation required the MYST domain of KAT8 and occurred at Lys^359^, within IRF3′s IAD. Unlike acetylation of IRF3 by p300, which promotes DNA binding, KAT8 selectively inhibited the recruitment of IRF3 to type-I IFN gene promoters, thus serving as a brake on virus-induced IFN production.

## 4. Viral Antagonism of IRF3: SARS-Coronavirus-Mediated Inhibition of IRF3 Activation

Severe acute respiratory syndrome-associated coronavirus (SARS-CoV) is an enveloped virus with a positive-sense single-stranded RNA genome that belongs to the family *Coronaviridae* [130]. It is the causative pathogen for the massive outbreak that infected thousands of people and claimed hundreds of lives in 2002 [131]. The coronavirus family is drawing attention again when a pandemic is sweeping over the entire world caused by another member of the same family, SARS-CoV-2 [132]. There are seven viruses within the coronavirus family known for their ability to cause severe disease in humans, while other members of this family cause only mild effects upon infection [133].

Recognition of virus by pathogen recognition receptors (PRRs) is essential in mounting host immune responses against viral infection. Studies in murine models have shown that both RIG-I and MDA5-dependent signaling pathways play important roles in activating downstream IRF3 and NF-κB in the defense against coronavirus infection [134]. While MDA5 is likely to recognize coronavirus replicating genome dsRNA products, RIG-I may be activated by coronavirus replication intermediates or negative sense subgenomic RNA [135]. It has been found that TLR3 recognition and subsequent signaling through TRIF play an important protective role in host immune response against SARS-CoV [136]. On one hand, recognition of coronavirus nucleic acids by PRRs could initiate various signaling pathways, activate IRF3, and induce IFN production and expression of ISGs. On the other hand, more evidence has shown that coronavirus could suppress the host IFN response through various mechanisms [137,138], a majority of which involve blocking the activation of IRF3 through inhibition of its upstream effectors [139] or direct interaction with IRF3 [140,141]. Transcriptional activation of IRF3 can be targeted by several viral proteins produced during coronavirus infection.

### 4.1. Antagonism of IRF3 by SARS-CoV Structural Proteins

**Figure 4:**
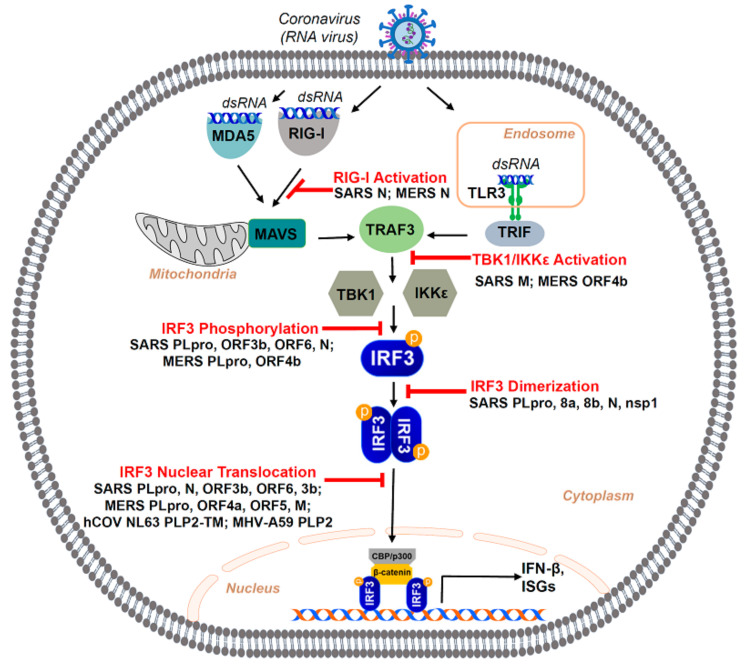
SARS-coronavirus (SARS-CoV) mediated inhibition of IRF3 activation. The SARS-CoV has various proteins that play a role in antagonizing IRF3 activation as a defense mechanism. Its nucleocapsid (N protein) acts by inhibiting RIG-I activation, one of the major PRRs responsible for IRF3 activation. Matrix (M) protein of SARS inhibits the activation of kinases (TBK-1, IKKε) required to activate IRF3. Various accessory proteins (Orf 3b, 6), protease (PLpro), as well as the N protein of the virus can antagonize the phosphorylation and activation of IRF3 itself. Even after IRF3 activation, SARS-CoV can use several accessory proteins (8a, 8b, 3b, 6) and non-structural proteins (nsp1) or the N-protein to block major changes, such as IRF3 dimerization or its nuclear translocation, that are essential for IRF3 to transcribe its downstream antiviral genes successfully. Some of these activation steps are also inhibited by MERS-CoV proteins, as shown in the cartoon.

There are four main structural proteins, envelope (E), membrane (M), spike (S), and nucleocapsid (N) glycoproteins, commonly produced by all known coronaviruses [142], studies of which have revealed their roles in antagonism of IRF3 (Figure 4).

The M (membrane) protein, also known as E1 membrane glycoprotein or matrix protein, is the most abundant structural protein and found to interact with all other structural proteins of coronaviruses [143]. The structural functions of M protein include binding with the N protein to stabilize the nucleocapsid and making a viral envelope together with E protein [143]. M protein has also been found to act as a potent IRF3 antagonist through various mechanisms. Researchers have found that SARS-CoV M protein blocked the formation of TRAF3·TANK·TBK1/IKKϵ complex and, therefore, inhibits the phosphorylation of IRF3 and production of IFN-β [144]. Interestingly, a recent paper presented the finding that M protein of SARS-CoV-2 is also capable of impeding IRF3 activation in a similar manner. SARS-CoV-2 M protein prevents the interaction between TBK1 and TRAF and inhibits the binding between RIG-I and MAVS, MAVS, and TBK1, thus interfering the formation of the RIG-I-MAVS-TRAF3-TBK1 multi-protein complex and inhibiting downstream IRF3 phosphorylation [145]. It is worth noting that the M protein of the Middle East respiratory syndrome (MERS), another member of coronavirus, physically interacts with TRAF to disrupt the TRAF–TBK1 interaction and, therefore, blocks the downstream activation and nuclear translocation of IRF3 as well as its binding to the promoter sites in the nucleus [146,147].

The nucleocapsid (N) protein is another structural protein of coronaviruses that could also function as an IRF3 antagonist through novel mechanisms. N Protein of SARS-CoV was shown to inhibit the activation and nuclear translocation of IRF3 induced by Poly I:C, a synthetic double-stranded RNA analog, and Sendai virus and is a potent antagonist of type-I IFN. However, the N protein does not inhibit IRF3 activation induced by MAVS, TRIF, TBK1, and IKKε. Further study demonstrated that N protein inhibits IRF3 activation and type-I IFN production in the early PRR recognition stage of the signaling pathway, and this is mainly achieved through binding the C terminal domain of N protein to the pathogenic nucleic acids and shielding the nucleic acids from recognition by host cell receptors [148]. A more recent study showed that SARS-CoV N protein also inhibits IRF3 activation and downstream type-I IFN production through suppression of RIG-I ubiquitination and activation. N Protein achieves this inhibition by binding its C terminus to the TRIM25 SPRY domain and, therefore, interfering with the TRIM25-RIG-I interaction and TRIM25 mediated RIG-I ubiquitination [149]. MERS-CoV N protein inhibits IRF3 in a similar fashion by blocking TRIM25 interaction with RIG-I and thus downregulating RIG-I signaling and downstream activation of IRF3 [150]. More studies need to be done to see whether the SARS-CoV-2 N protein shares the same IRF3 antagonistic ability.

### 4.2. Antagonism of IRF3 by SARS-CoV Non-Structural Proteins

The papain-like protease (PLpro) is an important coronavirus enzyme that processes viral polyproteins and promotes viral spread [141]. In 2007, Devaraj et al. demonstrated that the SARS-CoV papain-like protease (PLpro) domain of non-structural protein-3 (nsp3) in the viral genome can inhibit IRF3 through direct interaction to prevent IRF3 phosphorylation and nuclear translocation [141]. The binding of PLpro to IRF3 is mediated by its transmembrane domain, which disrupts the interaction of IRF3 with the upstream kinase TBK1 [141,151]. SARS-CoV PLpro and SARS-CoV-2 PLpro share a similar sequence identity; however, they possess different host substrate preferences. While SARS-CoV PLpro preferentially targets ubiquitin chains, SARS-CoV-2 PLpro mainly cleaves the ubiquitin-like interferon-stimulated gene 15 protein (ISG15) from IRF3 [152]. The cleavage of ISG15 from IRF3 by SARS-CoV-2 PLpro leads to the inhibition of type-I IFN responses. The PLpro of the MERS-CoV also blocks IRF3 phosphorylation and nuclear translocation [153]. Additionally, the non-structural protein 1 (nsp1) of SARS-CoV suppresses virus-dependent IRF3 phosphorylation and dimerization [154]. SARS-CoV-2 non-structural proteins nsp13, nsp14, and nsp15 inhibit IFN production upon RIG-I activation and dampen IRF3 nuclear translocation [155].

### 4.3. Antagonism of IRF3 by SARS-CoV Accessory Proteins

Accessory viral proteins possess activities that are not essential to viral replication but play roles in pathogenesis. The number, location, and size of accessory proteins could vary among different coronaviruses [156]. There are eight identified accessory proteins (3a, 3b, 6, 7a, 7b, 8a, 8b, and 9b) in SARS-CoV [157] and nine accessory proteins (3a, 3b, 6, 7a, 7b, 8, 9b, 9c, and 10) in SARS-CoV-2 [156]. Studies on the accessory proteins have provided increasing evidence that several of them could act as IRF3 antagonists. SARS-CoV open reading frames (Orf) 3b and 6 affect several steps of IRF3 activation, including IRF3 phosphorylation, nuclear translocation, and promoter binding [158]. SARS-CoV-2 Orf6 was shown to block IRF3 activation and nuclear translocation with its overexpression inhibiting RIG-I-like signaling pathways. Additionally, both SARS-CoV Orf6 and SARS-CoV-2 Orf6 showed similar attenuative effects on MAVS and IRF3-induced IFN-β-promoter activation via the C-terminus, which is critical for their antagonistic activity [159]. A 2017 study found that SARS-CoV accessory proteins 8b and 8ab physically interact with IRF3. In addition, the study showed that overexpression of 8b and 8ab decreases the dimerization of IRF3 and downstream type-I IFN production in Poly I:C treated cells. Moreover, it was discovered that proteins 8b and 8ab of SARS-CoV induce rapid degradation of IRF3 in a ubiquitin-dependent manner [140].

## 5. Clinical Implications of IRF3 Activation and Therapeutic Applications

The physiological relevance of IRF3 functions has primarily been evaluated using the knockout mice. The *Irf3^−/−^* mice are susceptible to a number of viral pathogens [48,160,161,162]. Similarly, the clinical relevance of IRF3 functions has been studied using genetic analyses of inborn errors in humans [163,164]. A heterozygous mutation of IRF3 (R258Q) in a patient is associated with functional IRF3 deficiencies that led to herpes simplex encephalitis [165,166]. These genetic analyses further strengthened the biochemical role of the positively-charged residue of IRF3 in its transcriptional activation [37]. A recent study suggested that an inborn error in the IRF3 gene is also associated with the susceptibility to SARS-CoV-2 infection [167]. The functional deficiency in IRF3 has also been evaluated in patients with mutations in proteins that activate IRF3. Patients with mutations in TLR3 have been shown to be associated with HSE [165], COVID-19 [168], and influenza pneumonitis [169]. Similarly, defects in RIG-I and RNA polymerase III are associated with the susceptibility to VZV [170] and IAV [171] infection. Deficiency in IRF7, which is activated similar to IRF3, has also been connected with the susceptibility to SARS-CoV-2 [168] and IAV [172] infections in humans.

Because IRF3 activation pathways are critical in human diseases, small molecule regulators of these pathways have been evaluated for potential therapeutic applications. A small molecule, CG-18, triggers the formation of IFNβ-enhanceosome by activating IRF3, NF-κB, and ATF2/c-Jun [173]. Small molecule activators of the RLR-IRF3 transcriptional pathway exhibit antiviral activities against many viruses, e.g., the flaviviruses (West Nile virus, dengue virus, hepatitis C virus), Ebola virus, IAV, Lassa virus, respiratory syncytial virus, and Nipah virus [174,175]. Activators of the RIG-I/IRF3 signaling pathway by a small molecule F7 or poly-U/UC PAMP lead to the suppression of covalently closed circular DNA formation by hepatitis B virus [176]. A small-molecule agonist of IRF3 has been shown to function as an influenza vaccine adjuvant by modulating the innate antiviral response [177]. In addition to the transcriptional activators of IRF3, we recently showed that the activation of the pro-apoptotic pathway of IRF3 also leads to cellular antiviral effects [60]. Therefore, therapeutic agents that activate IRF3 may be potential candidates for future antiviral drugs.

## 6. Conclusions

Posttranslational modifications of IRF3 by cellular and viral proteins are critical for the outcome of the host-virus interaction. Whether inactive IRF3 is also posttranslationally modified to regulate the cellular homeostasis is not clear. The discovery of non-transcriptional functions for IRF3 opens up new research areas to investigate the roles of IRFs in non-viral models. Whether other IRFs also exhibit non-transcriptional activities would be a potential future research direction. During the current state of the COVID-19 pandemic, the significance of non-transcriptional IRF3 remains to be determined. Because viruses, e.g., SARS-CoV-2, rapidly inhibit the transcriptional functions, ways to divert pools of IRF3 towards RIPA may provide new therapeutic alternatives. The drug screening may provide options to activate IRF3 in one pathway and not another to contribute to the overall antiviral response. The COVID-19 patients should be carefully examined for RIPA signatures in the early phases and sites of infection. The use of RIPA-promoters or caspase-activators may be an alternative therapeutic strategy. Given some autophagy inhibitors block SARS-CoV-2 replication [178], it is intriguing to speculate that these agents may block virus replication by promoting the apoptotic pathways. Because viruses and hosts have co-evolved, the viruses are beginning to turn to the RIPA-side of IRF3, as recently shown by Hardy et al. [179]. Whether such tug-of-war is applicable to SARS-CoV-2 will require in-depth investigation.

## Data Availability

Not applicable.

## References

[1] Marsili G., Perrotti E., Remoli A.L., Acchioni C., Sgarbanti M., Battistini A. (2016). IFN Regulatory Factors and Antiviral Innate Immunity: How Viruses Can Get Better. J. Interferon Cytokine Res..

[2] Jefferies C.A. (2019). Regulating IRFs in IFN Driven Disease. Front. Immunol..

[3] Ozato K., Tailor P., Kubota T. (2007). The interferon regulatory factor family in host defense: Mechanism of action. J. Biol. Chem..

[4] Hiscott J. (2007). Triggering the innate antiviral response through IRF-3 activation. J. Biol. Chem..

[5] Petro T.M. (2020). IFN Regulatory Factor 3 in Health and Disease. J. Immunol..

[6] Ning S., Pagano J.S., Barber G.N. (2011). IRF7: Activation, regulation, modification and function. Genes Immun..

[7] Thompson M.R., Kaminski J.J., Kurt-Jones E.A., Fitzgerald K.A. (2011). Pattern recognition receptors and the innate immune response to viral infection. Viruses.

[8] Brennan K., Bowie A.G. (2010). Activation of host pattern recognition receptors by viruses. Curr. Opin. Microbiol..

[9] Chattopadhyay S., Sen G.C. (2017). RIG-I-like receptor-induced IRF3 mediated pathway of apoptosis (RIPA): A new antiviral pathway. Protein Cell.

[10] Escalante C.R., Yie J., Thanos D., Aggarwal A.K. (1998). Structure of IRF-1 with bound DNA reveals determinants of interferon regulation. Nature.

[11] Takahasi K., Suzuki N.N., Horiuchi M., Mori M., Suhara W., Okabe Y., Fukuhara Y., Terasawa H., Akira S., Fujita T. (2003). X-ray crystal structure of IRF-3 and its functional implications. Nat. Struct. Biol..

[12] Qin B.Y., Liu C., Lam S.S., Srinath H., Delston R., Correia J.J., Derynck R., Lin K. (2003). Crystal structure of IRF-3 reveals mechanism of autoinhibition and virus-induced phosphoactivation. Nat. Struct. Biol..

[13] Karpova A.Y., Ronco L.V., Howley P.M. (2001). Functional characterization of interferon regulatory factor 3a (IRF-3a), an alternative splice isoform of IRF-3. Mol. Cell Biol..

[14] Takeuchi O., Akira S. (2010). Pattern recognition receptors and inflammation. Cell.

[15] Ablasser A., Bauernfeind F., Hartmann G., Latz E., Fitzgerald K.A., Hornung V. (2009). RIG-I-dependent sensing of poly(dA:dT) through the induction of an RNA polymerase III-transcribed RNA intermediate. Nat. Immunol..

[16] Ablasser A., Schmid-Burgk J.L., Hemmerling I., Horvath G.L., Schmidt T., Latz E., Hornung V. (2013). Cell intrinsic immunity spreads to bystander cells via the intercellular transfer of cGAMP. Nature.

[17] Satoh T., Akira S. (2016). Toll-Like Receptor Signaling and Its Inducible Proteins. Microbiol. Spectr..

[18] Kawasaki T., Kawai T. (2014). Toll-like receptor signaling pathways. Front. Immunol..

[19] Bermejo-Jambrina M., Eder J., Helgers L.C., Hertoghs N., Nijmeijer B.M., Stunnenberg M., Geijtenbeek T.B.H. (2018). C-Type Lectin Receptors in Antiviral Immunity and Viral Escape. Front. Immunol..

[20] Dambuza I.M., Brown G.D. (2015). C-type lectins in immunity: Recent developments. Curr. Opin. Immunol..

[21] Geijtenbeek T.B., Kwon D.S., Torensma R., van Vliet S.J., van Duijnhoven G.C., Middel J., Cornelissen I.L., Nottet H.S., KewalRamani V.N., Littman D.R. (2000). DC-SIGN, a dendritic cell-specific HIV-1-binding protein that enhances trans-infection of T cells. Cell.

[22] Moris A., Nobile C., Buseyne F., Porrot F., Abastado J.P., Schwartz O. (2004). DC-SIGN promotes exogenous MHC-I-restricted HIV-1 antigen presentation. Blood.

[23] Schlee M. (2013). Master sensors of pathogenic RNA—RIG-I like receptors. Immunobiology.

[24] Fensterl V., Chattopadhyay S., Sen G.C. (2015). No Love Lost Between Viruses and Interferons. Annu. Rev. Virol..

[25] Saito T., Hirai R., Loo Y.M., Owen D., Johnson C.L., Sinha S.C., Akira S., Fujita T., Gale M. (2007). Regulation of innate antiviral defenses through a shared repressor domain in RIG-I and LGP2. Proc. Natl. Acad. Sci. USA.

[26] Sengoku T., Nureki O., Nakamura A., Kobayashi S., Yokoyama S. (2006). Structural basis for RNA unwinding by the DEAD-box protein Drosophila Vasa. Cell.

[27] Brubaker S.W., Bonham K.S., Zanoni I., Kagan J.C. (2015). Innate immune pattern recognition: A cell biological perspective. Annu. Rev. Immunol..

[28] Hornung V., Ellegast J., Kim S., Brzozka K., Jung A., Kato H., Poeck H., Akira S., Conzelmann K.K., Schlee M. (2006). 5′-Triphosphate RNA is the ligand for RIG-I. Science.

[29] Fujita T. (2006). Virology. Sensing viral RNA amid your own. Science.

[30] Kawai T., Akira S. (2009). The roles of TLRs, RLRs and NLRs in pathogen recognition. Int. Immunol..

[31] Broquet A.H., Hirata Y., McAllister C.S., Kagnoff M.F. (2011). RIG-I/MDA5/MAVS are required to signal a protective IFN response in rotavirus-infected intestinal epithelium. J. Immunol..

[32] Horner S.M., Liu H.M., Park H.S., Briley J., Gale M. (2011). Mitochondrial-associated endoplasmic reticulum membranes (MAM) form innate immune synapses and are targeted by hepatitis C virus. Proc. Natl. Acad. Sci. USA.

[33] Kumar H., Kawai T., Kato H., Sato S., Takahashi K., Coban C., Yamamoto M., Uematsu S., Ishii K.J., Takeuchi O. (2006). Essential role of IPS-1 in innate immune responses against RNA viruses. J. Exp. Med..

[34] Sharma S., Grandvaux N., Zhou G.P., Lin R., Hiscott J. (2003). Triggering the interferon antiviral response through an IKK-related pathway. Science.

[35] Fitzgerald K.A., McWhirter S.M., Faia K.L., Rowe D.C., Latz E., Golenbock D.T., Coyle A.J., Liao S.M., Maniatis T. (2003). IKKepsilon and TBK1 are essential components of the IRF3 signaling pathway. Nat. Immunol..

[36] Xia P., Wang S., Gao P., Gao G., Fan Z. (2016). DNA sensor cGAS-mediated immune recognition. Protein Cell.

[37] Dalskov L., Narita R., Andersen L.L., Jensen N., Assil S., Kristensen K.H., Mikkelsen J.G., Fujita T., Mogensen T.H., Paludan S.R. (2020). Characterization of distinct molecular interactions responsible for IRF3 and IRF7 phosphorylation and subsequent dimerization. Nucleic Acids Res..

[38] Liu S., Cai X., Wu J., Cong Q., Chen X., Li T., Du F., Ren J., Wu Y.T., Grishin N.V. (2015). Phosphorylation of innate immune adaptor proteins MAVS, STING, and TRIF induces IRF3 activation. Science.

[39] Eckner R., Ewen M.E., Newsome D., Gerdes M., DeCaprio J.A., Lawrence J.B., Livingston D.M. (1994). Molecular cloning and functional analysis of the adenovirus E1A-associated 300-kD protein (p300) reveals a protein with properties of a transcriptional adaptor. Genes Dev..

[40] Yoneyama M., Suhara W., Fukuhara Y., Fukuda M., Nishida E., Fujita T. (1998). Direct triggering of the type I interferon system by virus infection: Activation of a transcription factor complex containing IRF-3 and CBP/p300. EMBO J..

[41] Chattopadhyay S., Fensterl V., Zhang Y., Veleeparambil M., Wetzel J.L., Sen G.C. (2013). Inhibition of viral pathogenesis and promotion of the septic shock response to bacterial infection by IRF-3 are regulated by the acetylation and phosphorylation of its coactivators. mBio.

[42] Zhu J., Coyne C.B., Sarkar S.N. (2011). PKC alpha regulates Sendai virus-mediated interferon induction through HDAC6 and β-catenin. EMBO J..

[43] Suhara W., Yoneyama M., Kitabayashi I., Fujita T. (2002). Direct involvement of CREB-binding protein/p300 in sequence-specific DNA binding of virus-activated interferon regulatory factor-3 holocomplex. J. Biol. Chem..

[44] Subramanian G., Kuzmanovic T., Zhang Y., Peter C.B., Veleeparambil M., Chakravarti R., Sen G.C., Chattopadhyay S. (2018). A new mechanism of interferon’s antiviral action: Induction of autophagy, essential for paramyxovirus replication, is inhibited by the interferon stimulated gene, TDRD7. PLoS Pathog..

[45] Cohen B., Novick D., Barak S., Rubinstein M. (1995). Ligand-induced association of the type I interferon receptor components. Mol. Cell Biol..

[46] Bandyopadhyay S.K., Leonard G.T., Bandyopadhyay T., Stark G.R., Sen G.C. (1995). Transcriptional induction by double-stranded RNA is mediated by interferon-stimulated response elements without activation of interferon-stimulated gene factor 3. J. Biol. Chem..

[47] Chattopadhyay S., Fensterl V., Zhang Y., Veleeparambil M., Yamashita M., Sen G.C. (2013). Role of interferon regulatory factor 3-mediated apoptosis in the establishment and maintenance of persistent infection by Sendai virus. J. Virol..

[48] Chattopadhyay S., Kuzmanovic T., Zhang Y., Wetzel J.L., Sen G.C. (2016). Ubiquitination of the Transcription Factor IRF-3 Activates RIPA, the Apoptotic Pathway that Protects Mice from Viral Pathogenesis. Immunity.

[49] Chattopadhyay S., Marques J.T., Yamashita M., Peters K.L., Smith K., Desai A., Williams B.R., Sen G.C. (2010). Viral apoptosis is induced by IRF-3-mediated activation of Bax. EMBO J..

[50] Peters K., Chattopadhyay S., Sen G.C. (2008). IRF-3 activation by Sendai virus infection is required for cellular apoptosis and avoidance of persistence. J. Virol..

[51] Chattopadhyay S., Yamashita M., Zhang Y., Sen G.C. (2011). The IRF-3/Bax-mediated apoptotic pathway, activated by viral cytoplasmic RNA and DNA, inhibits virus replication. J. Virol..

[52] White C.L., Chattopadhyay S., Sen G.C. (2011). Phosphatidylinositol 3-kinase signaling delays sendai virus-induced apoptosis by preventing XIAP degradation. J. Virol..

[53] Manzur M., Fleming P., Huang D.C., Degli-Esposti M.A., Andoniou C.E. (2009). Virally mediated inhibition of Bax in leukocytes promotes dissemination of murine cytomegalovirus. Cell Death Differ..

[54] Sze A., Belgnaoui S.M., Olagnier D., Lin R., Hiscott J., van Grevenynghe J. (2013). Host restriction factor SAMHD1 limits human T cell leukemia virus type 1 infection of monocytes via STING-mediated apoptosis. Cell Host Microbe.

[55] Zierhut C., Yamaguchi N., Paredes M., Luo J.D., Carroll T., Funabiki H. (2019). The Cytoplasmic DNA Sensor cGAS Promotes Mitotic Cell Death. Cell.

[56] Petrasek J., Iracheta-Vellve A., Csak T., Satishchandran A., Kodys K., Kurt-Jones E.A., Fitzgerald K.A., Szabo G. (2013). STING-IRF3 pathway links endoplasmic reticulum stress with hepatocyte apoptosis in early alcoholic liver disease. Proc. Natl. Acad. Sci. USA.

[57] Iracheta-Vellve A., Petrasek J., Gyongyosi B., Satishchandran A., Lowe P., Kodys K., Catalano D., Calenda C.D., Kurt-Jones E.A., Fitzgerald K.A. (2016). Endoplasmic Reticulum Stress-induced Hepatocellular Death Pathways Mediate Liver Injury and Fibrosis via Stimulator of Interferon Genes. J. Biol. Chem..

[58] Qiao J.T., Cui C., Qing L., Wang L.S., He T.Y., Yan F., Liu F.Q., Shen Y.H., Hou X.G., Chen L. (2018). Activation of the STING-IRF3 pathway promotes hepatocyte inflammation, apoptosis and induces metabolic disorders in nonalcoholic fatty liver disease. Metabolism.

[59] Sanz-Garcia C., McMullen M.R., Chattopadhyay S., Roychowdhury S., Sen G., Nagy L.E. (2019). Nontranscriptional Activity of Interferon Regulatory Factor 3 Protects Mice From High-Fat Diet-Induced Liver Injury. Hepatol. Commun..

[60] Glanz A., Chawla K., Fabry S., Subramanian G., Garcia J., Jay B., Ciricillo J., Chakravarti R., Taylor R.T., Chattopadhyay S. (2020). High Throughput Screening of FDA-Approved Drug Library Reveals the Compounds that Promote IRF3-Mediated Pro-Apoptotic Pathway Inhibit Virus Replication. Viruses.

[61] Vogel O.A., Han J., Liang C.Y., Manicassamy S., Perez J.T., Manicassamy B. (2020). The p150 Isoform of ADAR1 Blocks Sustained RLR signaling and Apoptosis during Influenza Virus Infection. PLoS Pathog..

[62] Sanz-Garcia C., Poulsen K.L., Bellos D., Wang H., McMullen M.R., Li X., Chattopadhyay S., Sen G., Nagy L.E. (2019). The non-transcriptional activity of IRF3 modulates hepatic immune cell populations in acute-on-chronic ethanol administration in mice. J. Hepatol..

[63] Kumar K.P., McBride K.M., Weaver B.K., Dingwall C., Reich N.C. (2000). Regulated nuclear-cytoplasmic localization of interferon regulatory factor 3, a subunit of double-stranded RNA-activated factor 1. Mol. Cell Biol..

[64] Lin R., Mamane Y., Hiscott J. (1999). Structural and functional analysis of interferon regulatory factor 3: Localization of the transactivation and autoinhibitory domains. Mol. Cell Biol..

[65] Chen W., Srinath H., Lam S.S., Schiffer C.A., Royer W.E., Lin K. (2008). Contribution of Ser386 and Ser396 to activation of interferon regulatory factor 3. J. Mol. Biol..

[66] Panne D., McWhirter S.M., Maniatis T., Harrison S.C. (2007). Interferon regulatory factor 3 is regulated by a dual phosphorylation-dependent switch. J. Biol. Chem..

[67] Bergstroem B., Johnsen I.B., Nguyen T.T., Hagen L., Slupphaug G., Thommesen L., Anthonsen M.W. (2010). Identification of a novel in vivo virus-targeted phosphorylation site in interferon regulatory factor-3 (IRF3). J. Biol. Chem..

[68] Clément J.F., Bibeau-Poirier A., Gravel S.P., Grandvaux N., Bonneil E., Thibault P., Meloche S., Servant M.J. (2008). Phosphorylation of IRF-3 on Ser 339 generates a hyperactive form of IRF-3 through regulation of dimerization and CBP association. J. Virol..

[69] Karpova A.Y., Trost M., Murray J.M., Cantley L.C., Howley P.M. (2002). Interferon regulatory factor-3 is an in vivo target of DNA-PK. Proc. Natl. Acad. Sci. USA.

[70] Luo F., Liu H., Yang S., Fang Y., Zhao Z., Hu Y., Jin Y., Li P., Gao T., Cao C. (2019). Nonreceptor Tyrosine Kinase c-Abl- and Arg-Mediated IRF3 Phosphorylation Regulates Innate Immune Responses by Promoting Type I IFN Production. J. Immunol..

[71] Li S., Zhu M., Pan R., Fang T., Cao Y.Y., Chen S., Zhao X., Lei C.Q., Guo L., Chen Y. (2016). The tumor suppressor PTEN has a critical role in antiviral innate immunity. Nat. Immunol..

[72] Meng F., Zhou R., Wu S., Zhang Q., Jin Q., Zhou Y., Plouffe S.W., Liu S., Song H., Xia Z. (2016). Mst1 shuts off cytosolic antiviral defense through IRF3 phosphorylation. Genes Dev..

[73] Wang J.T., Chang L.S., Chen C.J., Doong S.L., Chang C.W., Chen M.R. (2014). Glycogen synthase kinase 3 negatively regulates IFN regulatory factor 3 transactivation through phosphorylation at its linker region. Innate Immun..

[74] Wang J.T., Doong S.L., Teng S.C., Lee C.P., Tsai C.H., Chen M.R. (2009). Epstein-Barr virus BGLF4 kinase suppresses the interferon regulatory factor 3 signaling pathway. J. Virol..

[75] Saitoh T., Tun-Kyi A., Ryo A., Yamamoto M., Finn G., Fujita T., Akira S., Yamamoto N., Lu K.P., Yamaoka S. (2006). Negative regulation of interferon-regulatory factor 3-dependent innate antiviral response by the prolyl isomerase Pin1. Nat. Immunol..

[76] Gao L., Wang L., Dai T., Jin K., Zhang Z., Wang S., Xie F., Fang P., Yang B., Huang H. (2018). Tumor-derived exosomes antagonize innate antiviral immunity. Nat. Immunol..

[77] Bhoj V.G., Chen Z.J. (2009). Ubiquitylation in innate and adaptive immunity. Nature.

[78] Jiang X., Chen Z.J. (2011). The role of ubiquitylation in immune defence and pathogen evasion. Nat. Rev. Immunol..

[79] Zhang Z., Wang D., Wang P., Zhao Y., You F. (2020). OTUD1 Negatively Regulates Type I IFN Induction by Disrupting Noncanonical Ubiquitination of IRF3. J. Immunol..

[80] Spit M., Rieser E., Walczak H. (2019). Linear ubiquitination at a glance. J. Cell Sci..

[81] Inn K.S., Gack M.U., Tokunaga F., Shi M., Wong L.Y., Iwai K., Jung J.U. (2011). Linear ubiquitin assembly complex negatively regulates RIG-I- and TRIM25-mediated type I interferon induction. Mol. Cell.

[82] Rieser E., Cordier S.M., Walczak H. (2013). Linear ubiquitination: A newly discovered regulator of cell signalling. Trends Biochem. Sci..

[83] Kayagaki N., Phung Q., Chan S., Chaudhari R., Quan C., O’Rourke K.M., Eby M., Pietras E., Cheng G., Bazan J.F. (2007). DUBA: A deubiquitinase that regulates type I interferon production. Science.

[84] Sears N., Sen G.C., Stark G.R., Chattopadhyay S. (2011). Caspase-8-mediated cleavage inhibits IRF-3 protein by facilitating its proteasome-mediated degradation. J. Biol. Chem..

[85] Goswami R., Majumdar T., Dhar J., Chattopadhyay S., Bandyopadhyay S.K., Verbovetskaya V., Sen G.C., Barik S. (2013). Viral degradasome hijacks mitochondria to suppress innate immunity. Cell Res..

[86] Bibeau-Poirier A., Gravel S.P., Clement J.F., Rolland S., Rodier G., Coulombe P., Hiscott J., Grandvaux N., Meloche S., Servant M.J. (2006). Involvement of the IkappaB kinase (IKK)-related kinases tank-binding kinase 1/IKKi and cullin-based ubiquitin ligases in IFN regulatory factor-3 degradation. J. Immunol..

[87] Yu Y., Hayward G.S. (2010). The ubiquitin E3 ligase RAUL negatively regulates type i interferon through ubiquitination of the transcription factors IRF7 and IRF3. Immunity.

[88] Zhang M., Tian Y., Wang R.P., Gao D., Zhang Y., Diao F.C., Chen D.Y., Zhai Z.H., Shu H.B. (2008). Negative feedback regulation of cellular antiviral signaling by RBCK1-mediated degradation of IRF3. Cell Res..

[89] Higgs R., Ni Gabhann J., Ben Larbi N., Breen E.P., Fitzgerald K.A., Jefferies C.A. (2008). The E3 ubiquitin ligase Ro52 negatively regulates IFN-beta production post-pathogen recognition by polyubiquitin-mediated degradation of IRF3. J. Immunol..

[90] Rhodes D.A., Trowsdale J. (2007). TRIM21 is a trimeric protein that binds IgG Fc via the B30.2 domain. Mol. Immunol..

[91] Woo J.S., Imm J.H., Min C.K., Kim K.J., Cha S.S., Oh B.H. (2006). Structural and functional insights into the B30.2/SPRY domain. EMBO J..

[92] Wang P., Zhao W., Zhao K., Zhang L., Gao C. (2015). TRIM26 negatively regulates interferon-beta production and antiviral response through polyubiquitination and degradation of nuclear IRF3. PLoS Pathog..

[93] Qin Y., Zhou M.T., Hu M.M., Hu Y.H., Zhang J., Guo L., Zhong B., Shu H.B. (2014). RNF26 temporally regulates virus-triggered type I interferon induction by two distinct mechanisms. PLoS Pathog..

[94] Lei C.Q., Zhang Y., Xia T., Jiang L.Q., Zhong B., Shu H.B. (2013). FoxO1 negatively regulates cellular antiviral response by promoting degradation of IRF3. J. Biol. Chem..

[95] Litvak V., Ratushny A.V., Lampano A.E., Schmitz F., Huang A.C., Raman A., Rust A.G., Bergthaler A., Aitchison J.D., Aderem A. (2012). A FOXO3-IRF7 gene regulatory circuit limits inflammatory sequelae of antiviral responses. Nature.

[96] Zhao X., Zhu H., Yu J., Li H., Ge J., Chen W. (2016). c-Cbl-mediated ubiquitination of IRF3 negatively regulates IFN-beta production and cellular antiviral response. Cell. Signal..

[97] Xiong H., Li H., Kong H.J., Chen Y., Zhao J., Xiong S., Huang B., Gu H., Mayer L., Ozato K. (2005). Ubiquitin-dependent degradation of interferon regulatory factor-8 mediated by Cbl down-regulates interleukin-12 expression. J. Biol. Chem..

[98] Zeng W., Xu M., Liu S., Sun L., Chen Z.J. (2009). Key role of Ubc5 and lysine-63 polyubiquitination in viral activation of IRF3. Mol. Cell.

[99] Lu D., Song J., Sun Y., Qi F., Liu L., Jin Y., McNutt M.A., Yin Y. (2018). Mutations of deubiquitinase OTUD1 are associated with autoimmune disorders. J. Autoimmun..

[100] Zheng Y., Jayappa K.D., Ao Z., Qiu X., Su R.C., Yao X. (2019). Noncovalent SUMO-interaction motifs in HIV integrase play important roles in SUMOylation, cofactor binding, and virus replication. Virol. J..

[101] Hu M.M., Yang Q., Xie X.Q., Liao C.Y., Lin H., Liu T.T., Yin L., Shu H.B. (2016). Sumoylation Promotes the Stability of the DNA Sensor cGAS and the Adaptor STING to Regulate the Kinetics of Response to DNA Virus. Immunity.

[102] Kubota T., Matsuoka M., Chang T.H., Tailor P., Sasaki T., Tashiro M., Kato A., Ozato K. (2008). Virus infection triggers SUMOylation of IRF3 and IRF7, leading to the negative regulation of type I interferon gene expression. J. Biol. Chem..

[103] Ran Y., Liu T.T., Zhou Q., Li S., Mao A.P., Li Y., Liu L.J., Cheng J.K., Shu H.B. (2011). SENP2 negatively regulates cellular antiviral response by deSUMOylating IRF3 and conditioning it for ubiquitination and degradation. J. Mol. Cell Biol..

[104] Maarifi G., Hannoun Z., Geoffroy M.C., El Asmi F., Zarrouk K., Nisole S., Blondel D., Chelbi-Alix M.K. (2016). MxA Mediates SUMO-Induced Resistance to Vesicular Stomatitis Virus. J. Virol..

[105] Dalle-Donne I., Rossi R., Colombo G., Giustarini D., Milzani A. (2009). Protein S-glutathionylation: A regulatory device from bacteria to humans. Trends Biochem. Sci..

[106] Zhang J., Ye Z.W., Singh S., Townsend D.M., Tew K.D. (2018). An evolving understanding of the S-glutathionylation cycle in pathways of redox regulation. Free Radic. Biol. Med..

[107] Shelton M.D., Mieyal J.J. (2008). Regulation by reversible S-glutathionylation: Molecular targets implicated in inflammatory diseases. Mol. Cells.

[108] Prinarakis E., Chantzoura E., Thanos D., Spyrou G. (2008). S-glutathionylation of IRF3 regulates IRF3-CBP interaction and activation of the IFN beta pathway. EMBO J..

[109] Mowen K.A., David M. (2014). Unconventional post-translational modifications in immunological signaling. Nat. Immunol..

[110] Biggar K.K., Li S.S. (2015). Non-histone protein methylation as a regulator of cellular signalling and function. Nat. Rev. Mol. Cell Biol..

[111] Wang C., Wang Q., Xu X., Xie B., Zhao Y., Li N., Cao X. (2017). The methyltransferase NSD3 promotes antiviral innate immunity via direct lysine methylation of IRF3. J. Exp. Med..

[112] Morales D.J., Lenschow D.J. (2013). The antiviral activities of ISG15. J. Mol. Biol.

[113] Haas A.L., Ahrens P., Bright P.M., Ankel H. (1987). Interferon induces a 15-kilodalton protein exhibiting marked homology to ubiquitin. J. Biol. Chem..

[114] Dao C.T., Zhang D.E. (2005). ISG15: A ubiquitin-like enigma. Front. Biosci..

[115] Narasimhan J., Wang M., Fu Z., Klein J.M., Haas A.L., Kim J.J. (2005). Crystal structure of the interferon-induced ubiquitin-like protein ISG15. J. Biol. Chem..

[116] Zhao C., Denison C., Huibregtse J.M., Gygi S., Krug R.M. (2005). Human ISG15 conjugation targets both IFN-induced and constitutively expressed proteins functioning in diverse cellular pathways. Proc. Natl. Acad. Sci. USA.

[117] Arimoto K., Konishi H., Shimotohno K. (2008). UbcH8 regulates ubiquitin and ISG15 conjugation to RIG-I. Mol. Immunol..

[118] Giannakopoulos N.V., Luo J.K., Papov V., Zou W., Lenschow D.J., Jacobs B.S., Borden E.C., Li J., Virgin H.W., Zhang D.E. (2005). Proteomic identification of proteins conjugated to ISG15 in mouse and human cells. Biochem. Biophys. Res. Commun..

[119] Dastur A., Beaudenon S., Kelley M., Krug R.M., Huibregtse J.M. (2006). Herc5, an interferon-induced HECT E3 enzyme, is required for conjugation of ISG15 in human cells. J. Biol. Chem..

[120] Wong J.J., Pung Y.F., Sze N.S., Chin K.C. (2006). HERC5 is an IFN-induced HECT-type E3 protein ligase that mediates type I IFN-induced ISGylation of protein targets. Proc. Natl. Acad. Sci. USA.

[121] Shi H.X., Yang K., Liu X., Liu X.Y., Wei B., Shan Y.F., Zhu L.H., Wang C. (2010). Positive regulation of interferon regulatory factor 3 activation by Herc5 via ISG15 modification. Mol. Cell Biol..

[122] Phillips D.M. (1963). The presence of acetyl groups of histones. Biochem. J..

[123] Allfrey V.G., Faulkner R., Mirsky A.E. (1964). Acetylation and methylation of histones and their possible role in the regulation of RNA synthesis. Proc. Natl. Acad. Sci. USA.

[124] Caillaud A., Prakash A., Smith E., Masumi A., Hovanessian A.G., Levy D.E., Marie I. (2002). Acetylation of interferon regulatory factor-7 by p300/CREB-binding protein (CBP)-associated factor (PCAF) impairs its DNA binding. J. Biol. Chem..

[125] Wang Z., Zang C., Cui K., Schones D.E., Barski A., Peng W., Zhao K. (2009). Genome-wide mapping of HATs and HDACs reveals distinct functions in active and inactive genes. Cell.

[126] Tang X., Gao J.S., Guan Y.J., McLane K.E., Yuan Z.L., Ramratnam B., Chin Y.E. (2007). Acetylation-dependent signal transduction for type I interferon receptor. Cell.

[127] Hu X., Yu Y., Eugene Chin Y., Xia Q. (2013). The role of acetylation in TLR4-mediated innate immune responses. Immunol. Cell Biol..

[128] Masumi A., Ozato K. (2001). Coactivator p300 acetylates the interferon regulatory factor-2 in U937 cells following phorbol ester treatment. J. Biol. Chem..

[129] Huai W., Liu X., Wang C., Zhang Y., Chen X., Chen X., Xu S., Thomas T., Li N., Cao X. (2019). KAT8 selectively inhibits antiviral immunity by acetylating IRF3. J. Exp. Med..

[130] Perlman S., Netland J. (2009). Coronaviruses post-SARS: Update on replication and pathogenesis. Nat. Rev. Microbiol..

[131] Zhong N.S., Zheng B.J., Li Y.M., Poon L.L.M., Xie Z.H., Chan K.H., Li P.H., Tan S.Y., Chang Q., Xie J.P. (2003). Epidemiology and cause of severe acute respiratory syndrome (SARS) in Guangdong, People’s Republic of China, in February, 2003. Lancet.

[132] Golonka R.M., Saha P., Yeoh B.S., Chattopadhyay S., Gewirtz A.T., Joe B., Vijay-Kumar M. (2020). Harnessing innate immunity to eliminate SARS-CoV-2 and ameliorate COVID-19 disease. Physiol. Genom..

[133] Cui J., Li F., Shi Z.L. (2019). Origin and evolution of pathogenic coronaviruses. Nat. Rev. Microbiol..

[134] Li J., Liu Y., Zhang X. (2010). Murine coronavirus induces type I interferon in oligodendrocytes through recognition by RIG-I and MDA5. J. Virol..

[135] Kell A.M., Gale M. (2015). RIG-I in RNA virus recognition. Virology.

[136] Totura A.L., Whitmore A., Agnihothram S., Schäfer A., Katze M.G., Heise M.T., Baric R.S. (2015). Toll-Like Receptor 3 Signaling via TRIF Contributes to a Protective Innate Immune Response to Severe Acute Respiratory Syndrome Coronavirus Infection. mBio.

[137] Spiegel M., Pichlmair A., Martínez-Sobrido L., Cros J., García-Sastre A., Haller O., Weber F. (2005). Inhibition of Beta interferon induction by severe acute respiratory syndrome coronavirus suggests a two-step model for activation of interferon regulatory factor 3. J. Virol..

[138] Totura A.L., Baric R.S. (2012). SARS coronavirus pathogenesis: Host innate immune responses and viral antagonism of interferon. Curr. Opin. Virol..

[139] Clementz M.A., Chen Z., Banach B.S., Wang Y., Sun L., Ratia K., Baez-Santos Y.M., Wang J., Takayama J., Ghosh A.K. (2010). Deubiquitinating and interferon antagonism activities of coronavirus papain-like proteases. J. Virol..

[140] Wong H.H., Fung T.S., Fang S., Huang M., Le M.T., Liu D.X. (2018). Accessory proteins 8b and 8ab of severe acute respiratory syndrome coronavirus suppress the interferon signaling pathway by mediating ubiquitin-dependent rapid degradation of interferon regulatory factor 3. Virology.

[141] Devaraj S.G., Wang N., Chen Z., Chen Z., Tseng M., Barretto N., Lin R., Peters C.J., Tseng C.T., Baker S.C. (2007). Regulation of IRF-3-dependent innate immunity by the papain-like protease domain of the severe acute respiratory syndrome coronavirus. J. Biol. Chem..

[142] Rota P.A., Oberste M.S., Monroe S.S., Nix W.A., Campagnoli R., Icenogle J.P., Peñaranda S., Bankamp B., Maher K., Chen M.H. (2003). Characterization of a novel coronavirus associated with severe acute respiratory syndrome. Science.

[143] Schoeman D., Fielding B.C. (2019). Coronavirus envelope protein: Current knowledge. Virol. J..

[144] Siu K.L., Kok K.H., Ng M.H., Poon V.K., Yuen K.Y., Zheng B.J., Jin D.Y. (2009). Severe acute respiratory syndrome coronavirus M protein inhibits type I interferon production by impeding the formation of TRAF3.TANK.TBK1/IKKepsilon complex. J. Biol. Chem..

[145] Zheng Y., Zhuang M.-W., Han L., Zhang J., Nan M.-L., Gao C., Wang P.-H. (2020). Severe Acute Respiratory Syndrome Coronavirus 2 (SARS-CoV-2) Membrane (M) Protein Inhibits Type I and III Interferon Production by Targeting RIG-I/MDA-5 Signaling. bioRxiv.

[146] Lui P.Y., Wong L.Y., Fung C.L., Siu K.L., Yeung M.L., Yuen K.S., Chan C.P., Woo P.C., Yuen K.Y., Jin D.Y. (2016). Middle East respiratory syndrome coronavirus M protein suppresses type I interferon expression through the inhibition of TBK1-dependent phosphorylation of IRF3. Emerg. Microbes Infect..

[147] Yang Y., Zhang L., Geng H., Deng Y., Huang B., Guo Y., Zhao Z., Tan W. (2013). The structural and accessory proteins M, ORF 4a, ORF 4b, and ORF 5 of Middle East respiratory syndrome coronavirus (MERS-CoV) are potent interferon antagonists. Protein Cell.

[148] Lu X., Pan J., Tao J., Guo D. (2011). SARS-CoV nucleocapsid protein antagonizes IFN-β response by targeting initial step of IFN-β induction pathway, and its C-terminal region is critical for the antagonism. Virus Genes.

[149] Hu Y., Li W., Gao T., Cui Y., Jin Y., Li P., Ma Q., Liu X., Cao C. (2017). The Severe Acute Respiratory Syndrome Coronavirus Nucleocapsid Inhibits Type I Interferon Production by Interfering with TRIM25-Mediated RIG-I Ubiquitination. J. Virol..

[150] Chang C.Y., Liu H.M., Chang M.F., Chang S.C. (2020). Middle East respiratory syndrome coronavirus nucleocapsid protein suppresses type I and type III interferon induction by targeting RIG-I signaling. J. Virol..

[151] Chen X., Yang X., Zheng Y., Yang Y., Xing Y., Chen Z. (2014). SARS coronavirus papain-like protease inhibits the type I interferon signaling pathway through interaction with the STING-TRAF3-TBK1 complex. Protein Cell.

[152] Shin D., Mukherjee R., Grewe D., Bojkova D., Baek K., Bhattacharya A., Schulz L., Widera M., Mehdipour A.R., Tascher G. (2020). Papain-like protease regulates SARS-CoV-2 viral spread and innate immunity. Nature.

[153] Yang X., Chen X., Bian G., Tu J., Xing Y., Wang Y., Chen Z. (2014). Proteolytic processing, deubiquitinase and interferon antagonist activities of Middle East respiratory syndrome coronavirus papain-like protease. J. Gen. Virol..

[154] Wathelet M.G., Orr M., Frieman M.B., Baric R.S. (2007). Severe acute respiratory syndrome coronavirus evades antiviral signaling: Role of nsp1 and rational design of an attenuated strain. J. Virol..

[155] Yuen C.K., Lam J.Y., Wong W.M., Mak L.F., Wang X., Chu H., Cai J.P., Jin D.Y., To K.K., Chan J.F. (2020). SARS-CoV-2 nsp13, nsp14, nsp15 and orf6 function as potent interferon antagonists. Emerg. Microbes Infect..

[156] Michel C.J., Mayer C., Poch O., Thompson J.D. (2020). Characterization of accessory genes in coronavirus genomes. Virol. J..

[157] Narayanan K., Huang C., Makino S. (2008). SARS coronavirus accessory proteins. Virus Res..

[158] Kopecky-Bromberg S.A., Martínez-Sobrido L., Frieman M., Baric R.A., Palese P. (2007). Severe acute respiratory syndrome coronavirus open reading frame (ORF) 3b, ORF 6, and nucleocapsid proteins function as interferon antagonists. J. Virol..

[159] Lei X., Dong X., Ma R., Wang W., Xiao X., Tian Z., Wang C., Wang Y., Li L., Ren L. (2020). Activation and evasion of type I interferon responses by SARS-CoV-2. Nat. Commun..

[160] Sato M., Suemori H., Hata N., Asagiri M., Ogasawara K., Nakao K., Nakaya T., Katsuki M., Noguchi S., Tanaka N. (2000). Distinct and essential roles of transcription factors IRF-3 and IRF-7 in response to viruses for IFN-alpha/beta gene induction. Immunity.

[161] Chen H.W., King K., Tu J., Sanchez M., Luster A.D., Shresta S. (2013). The roles of IRF-3 and IRF-7 in innate antiviral immunity against dengue virus. J. Immunol..

[162] Lazear H.M., Lancaster A., Wilkins C., Suthar M.S., Huang A., Vick S.C., Clepper L., Thackray L., Brassil M.M., Virgin H.W. (2013). IRF-3, IRF-5, and IRF-7 coordinately regulate the type I IFN response in myeloid dendritic cells downstream of MAVS signaling. PLoS Pathog..

[163] Mogensen T.H. (2018). IRF and STAT Transcription Factors—From Basic Biology to Roles in Infection, Protective Immunity, and Primary Immunodeficiencies. Front. Immunol..

[164] Jorgensen S.E., Al-Mousawi A., Assing K., Hartling U., Grosen D., Fisker N., Nielsen C., Jakobsen M.A., Mogensen T.H. (2021). STK4 Deficiency Impairs Innate Immunity and Interferon Production Through Negative Regulation of TBK1-IRF3 Signaling. J. Clin. Immunol..

[165] Zhang S.Y., Casanova J.L. (2015). Inborn errors underlying herpes simplex encephalitis: From TLR3 to IRF3. J. Exp. Med..

[166] Andersen L.L., Mork N., Reinert L.S., Kofod-Olsen E., Narita R., Jorgensen S.E., Skipper K.A., Honing K., Gad H.H., Ostergaard L. (2015). Functional IRF3 deficiency in a patient with herpes simplex encephalitis. J. Exp. Med..

[167] Levy R., Bastard P., Lanternier F., Lecuit M., Zhang S.Y., Casanova J.L. (2021). IFN-alpha2a Therapy in Two Patients with Inborn Errors of TLR3 and IRF3 Infected with SARS-CoV-2. J. Clin. Immunol..

[168] Zhang Q., Bastard P., Liu Z., Le Pen J., Moncada-Velez M., Chen J., Ogishi M., Sabli I.K.D., Hodeib S., Korol C. (2020). Inborn errors of type I IFN immunity in patients with life-threatening COVID-19. Science.

[169] Lim H.K., Huang S.X.L., Chen J., Kerner G., Gilliaux O., Bastard P., Dobbs K., Hernandez N., Goudin N., Hasek M.L. (2019). Severe influenza pneumonitis in children with inherited TLR3 deficiency. J. Exp. Med..

[170] Ogunjimi B., Zhang S.Y., Sorensen K.B., Skipper K.A., Carter-Timofte M., Kerner G., Luecke S., Prabakaran T., Cai Y., Meester J. (2017). Inborn errors in RNA polymerase III underlie severe varicella zoster virus infections. J. Clin. Investig..

[171] Jorgensen S.E., Christiansen M., Ryo L.B., Gad H.H., Gjedsted J., Staeheli P., Mikkelsen J.G., Storgaard M., Hartmann R., Mogensen T.H. (2018). Defective RNA sensing by RIG-I in severe influenza virus infection. Clin. Exp. Immunol..

[172] Thomsen M.M., Jorgensen S.E., Gad H.H., Storgaard M., Gjedsted J., Christiansen M., Hartmann R., Mogensen T.H. (2019). Defective interferon priming and impaired antiviral responses in a patient with an IRF7 variant and severe influenza. Med. Microbiol. Immunol..

[173] Kim T., Kim T.Y., Lee W.G., Yim J., Kim T.K. (2000). Signaling pathways to the assembly of an interferon-beta enhanceosome. Chemical genetic studies with a small molecule. J. Biol. Chem..

[174] Pattabhi S., Wilkins C.R., Dong R., Knoll M.L., Posakony J., Kaiser S., Mire C.E., Wang M.L., Ireton R.C., Geisbert T.W. (2015). Targeting Innate Immunity for Antiviral Therapy through Small Molecule Agonists of the RLR Pathway. J. Virol..

[175] Green R.R., Wilkins C., Pattabhi S., Dong R., Loo Y., Gale M. (2016). Transcriptional analysis of antiviral small molecule therapeutics as agonists of the RLR pathway. Genom. Data.

[176] Lee S., Goyal A., Perelson A.S., Ishida Y., Saito T., Gale M. (2021). Suppression of hepatitis B virus through therapeutic activation of RIG-I and IRF3 signaling in hepatocytes. iScience.

[177] Probst P., Grigg J.B., Wang M., Munoz E., Loo Y.M., Ireton R.C., Gale M., Iadonato S.P., Bedard K.M. (2017). A small-molecule IRF3 agonist functions as an influenza vaccine adjuvant by modulating the antiviral immune response. Vaccine.

[178] Bonam S.R., Muller S., Bayry J., Klionsky D.J. (2020). Autophagy as an emerging target for COVID-19: Lessons from an old friend, chloroquine. Autophagy.

[179] Hardy S., Jackson B., Goodbourn S., Seago J. (2020). Classical swine fever virus N(pro) antagonises IRF3 to prevent IFN-independent TLR3 and RIG-I-mediated apoptosis. J. Virol..

